# Genome-wide identification and characterization of cacao WRKY transcription factors and analysis of their expression in response to witches' broom disease

**DOI:** 10.1371/journal.pone.0187346

**Published:** 2017-10-30

**Authors:** Dayanne Silva Monteiro de Almeida, Daniel Oliveira Jordão do Amaral, Luiz-Eduardo Del-Bem, Emily Bronze dos Santos, Raner José Santana Silva, Karina Peres Gramacho, Michel Vincentz, Fabienne Micheli

**Affiliations:** 1 Universidade Estadual de Santa Cruz (UESC), Departamento de Ciências Biológicas (DCB), Centro de Biotecnologia e Genética (CBG), Rodovia Ilhéus-Itabuna, km 16, Ilhéus-BA, Brazil; 2 Centro de Biologia Molecular e Engenharia Genética, Departamento de Genética e Evolução, Instituto de Biologia, Universidade Estadual de Campinas, Campinas, Brasil; 3 Cocoa Research Center, CEPLAC/CEPEC, Itabuna-BA, Brazil; 4 CIRAD, UMR AGAP, Montpellier, France; McGill University, CANADA

## Abstract

Transcriptional regulation, led by transcription factors (TFs) such as those of the WRKY family, is a mechanism used by the organism to enhance or repress gene expression in response to stimuli. Here, we report on the genome-wide analysis of the *Theobroma cacao* WRKY TF family and also investigate the expression of WRKY genes in cacao infected by the fungus *Moniliophthora perniciosa*. In the cacao genome, 61 non-redundant WRKY sequences were found and classified in three groups (I to III) according to the WRKY and zinc-finger motif types. The 61 putative WRKY sequences were distributed on the 10 cacao chromosomes and 24 of them came from duplication events. The sequences were phylogenetically organized according to the general WRKY groups. The phylogenetic analysis revealed that subgroups IIa and IIb are sister groups and share a common ancestor, as well as subgroups IId and IIe. The most divergent groups according to the plant origin were IIc and III. According to the phylogenetic analysis, 7 *TcWRKY* genes were selected and analyzed by RT-qPCR in susceptible and resistant cacao plants infected (or not) with *M*. *perniciosa*. Some *TcWRKY* genes presented interesting responses to *M*. *perniciosa* such as Tc01_p014750/Tc06_p013130/AtWRKY28, Tc09_p001530/Tc06_p004420/AtWRKY40, Tc04_p016130/AtWRKY54 and Tc10_p016570/ AtWRKY70. Our results can help to select appropriate candidate genes for further characterization in cacao or in other *Theobroma* species.

## Introduction

Plants, whether growing under natural or agricultural conditions, are exposed to adverse environmental situations that affect their development and can drastically reduce their productivity. Such environmental stimuli can be abiotic (e.g., drought, cold, wounds) or caused by pathogens [1]. However, plants have mechanisms to survive in such adverse conditions, including tolerance or resistance to stress through adaptation mechanisms [2]. When stress conditions are detected by the plants, complex transduction pathways are induced, initiating a series of molecular, physiological and metabolic events, generally leading to an increase of tolerance/resistance [3]. Transcriptional regulation, led by transcription factors (TFs)–regulation proteins that link DNA sequences in specific promoter regions of target genes–is the first mechanism that is activated by the organism to enhance or repress gene expression involved in response to internal or external stimuli [4]. Such TFs can regulate more than one gene as well as other TFs [4].

Among TFs, the WRKY family is well known. The first WRKY protein was identified in sweet potato [5] and since then, WRKY proteins have been characterized in several other plants [6, 7] as well as in algae and non-plant eukaryotes [8]. The WRKY TFs bind a specific promoter sequence of the target gene, known as a W-box, positively or negatively regulating the target gene expression. The WRKY proteins contain one or two DNA binding domains of 60 amino acids containing the conserved heptapeptide WRKYGQK followed by a zinc-finger motif C_2_H_2_ (C-X_4-5_-C-X_22-23_-H-X-H) or C_2_HC (C-X_7_-C-X_23-24_-H-X-C) [6]. In some species, members of the WRKY family containing three DNA binding domains have been found [9, 10]. The WRKY TF family is known to be involved in response to biotic and abiotic stresses [7], and also to modulate various other processes in plants such as embryogenesis [11], trichome and seed development [12], leaf senescence [13], fruit and pollen development [14], biomass [15], secondary metabolite biosynthesis [16] and hormone signalling [17]. In *Arabidopsis* species, several *WRKY* genes have been experimentally characterized and associated with response to fungal or bacterial pathogens [18, 19], as well as to nematodes [20]. In cacao (*Theobroma cacao* L.), a portion of *WRKY* genes have been previously identified and analyzed with regards to their expression, phylogeny within the Malvaceae family, and/or for their potential use in marker assisted selection [21–23], but no exhaustive analysis of this TF family based on cacao genome data has been carried out.

The cacao tree is cultivated mainly for its beans, used as raw material for making chocolate. However, bean production is threatened worldwide by several pathogens, such as *M*. *perniciosa*, the agent of the witches’ broom disease [24], one of the most devastating diseases of the crop in Central and South America, and the Caribbean [25]. This disease has been responsible for the abandonment by producers of many cultivated areas in these regions [26], to the point of causing a shortage of cacao beans in the global market [27]. In this scenario, during the genomic and post-genomic eras, tools have been developed to understand the cacao-*M*. *perniciosa* interaction as well as to identify molecules that can be used to develop disease control methods. Here, we report on the genome-wide analysis of the *T*. *cacao* WRKY TF family and the identification of a comprehensive and non-redundant set of *WRKY* genes from this species. Subsequently, chromosomal location was determined and phylogenetic and motif analyses performed as a base for further comparative genomics studies. Moreover, expression patterns of *WRKY* genes in cacao infected (or not) with the pathogenic fungus *M*. *perniciosa* were also investigated. From the 61 TcWRKY proteins identified some were potentially involved cacao’s response to *M*. *perniciosa* and can be considered good candidates for subsequent functional analyses or disease management.

## Material and methods

### Datasets and WRKY protein identification

The scheme of the *in silico* pipeline used for identification of the *Theobroma cacao* WRKY protein set is shown in the S1 Fig. The *Theobroma cacao* protein sequences were downloaded from the CocoaGenDB database v1.0 (*Theobroma*_*cacao*_v1.pep.faa.gz; http://cocoa-genome-hub.southgreen.fr/gbrowse) [28]. The WRKY protein sequences of *Arabidopsis thaliana* were downloaded from the Phytozome database v12.1 (www.phytozome.org) and plant transcription factor database v.3.0 (http://plntfdb.bio.uni-potsdam.de/v3.0); only the non-redundant proteins between both databases were considered and used for the subsequent analysis (S1 Fig). The cacao proteins were screened to search for WRKY members using the local BLASTP program (blastall version 2.2.27; ftp://ftp.ncbi.nih.gov/blast/executables/blast+/LATEST) and the WRKY protein sequences of *A*. *thaliana* as input sequences (S1 Fig). The e-value for BLASTP was set at 1e-10 to obtain the final dataset of WRKY proteins. When more than one alternative splicing sequence was found for the same locus, only the longest non-redundant sequences were used for subsequent analyses. Subsequently, these candidate TcWRKY protein sequences were submitted to analysis using the InterPro (http://www.ebi.ac.uk/interpro/) and PFAM programs (http://pfam.xfam.org/) to confirm the presence of the WRKY domain, and analyzed using the SMART program (http://smart.embl-heidelberg.de/) to confirm the presence of the zinc-finger domain (S1 Fig). The protein sequences lacking both the WRKY and the zinc-finger domains were manually excluded. In parallel, an automatic search of TcWRKY proteins was made using the browser tool of CocoaGenDB v.1.0 based on keywords and InterPro numbers. The comparison of results from both analyses (local BlastP *vs* automatic search) allowed a fine, precise and complete analysis guaranteeing the identification of the largest non-redundant TcWRKY TF set (S1 Fig). This final largest non-redundant TcWRKY TF set contained 61 proteins (Table 1). Two of the TcWRKY proteins presented a highly altered WRKY motif and/or zinc-finger motif (Tc00_g017240 and Tc02_g001170) and for this reason were excluded from the phylogenetic analysis (Table 1). Moreover, one of the TcWRKY proteins presented the zinc-finger motif but not the conserved heptapeptide (Tc02_g012180) for this reason was excluded from all analyses (Table 1). Thus, depending on the analysis, only 60 or 58 protein sequences were used.

**Table 1:** WRKY proteins present in the cacao genome (CacaoGenDB v1.0 [28]). The variants of the conserved WRKYGQK peptide are shown in bold. nd: not determined.

Gene locus	WRKY domain			Group	Chromosome	Acession number
	Conserved heptapeptide	Zinc-finger type	Domain amount			
Tc02_g032670	WRKYGQK/ WRKYGQK	C_2_H_2_	2	I	2	XP_007045981.1
Tc04_g009710	WRKYGQK/ WRKYGQK	C_2_H_2_	2	I	4	XP_007032626.1
Tc09_g034740	WRKYGQK/ WRKYGQK	C_2_H_2_	2	I	9	XP_007017012.1
Tc05_g001480	WRKYGQK/ WRKYGQK	C_2_H_2_	2	I	5	XP_007026547.1
Tc05_g005710	WRKYGQK/ WRKYGQK	C_2_H_2_	2	I	5	XP_007027196.1
Tc05_g020810	WRKYGQK/ WRKYGQK	C_2_H_2_	2	I	5	XP_007029376.1
Tc07_g002020	WRKYGQK/ WRKYGQK	C_2_H_2_	2	I	7	XP_007020620.1
Tc07_g000190	WRKYGQK/ WRKYGQK	C_2_H_2_	2	I	7	XP_007020301.1
Tc01_g018460	WRKYGQK/ WRKYGQK	C_2_H_2_	2	I	1	XP_007049282.1
Tc09_g002780	WRKYGQK/ WRKYGQK	C_2_H_2_	2	I	9	XP_007011940.1
Tc09_g001530	WRKYGQK	C_2_H_2_	1	IIa	9	XP_007011727.1
Tc09_g001520	WRKYGQK	C_2_H_2_	1	IIa	9	XP_007011726.1
Tc06_g004420	WRKYGQK	C_2_H_2_	1	IIa	6	XP_007023430.1
Tc06_g019530	WRKYGQK	C_2_H_2_	1	IIb	6	XP_007026134.1
Tc02_g003250	WRKYGQK	C_2_H_2_	1	IIb	2	XP_007041570.1
Tc01_g032940	WRKYGQK	C_2_H_2_	1	IIb	1	XP_007051308.1
Tc01_g017430	WRKYGQK	C_2_H_2_	1	IIb	1	XP_007049086.1
Tc03_g009820	WRKYGQK	C_2_H_2_	1	IIb	3	XP_007037253.1
Tc07_g002910	WRKYGQK	C_2_H_2_	1	IIb	7	XP_007020766.1
Tc04_g007790	WRKYGQK	C_2_H_2_	1	IIb	4	XP_007032468.1
Tc02_g033950	WRKYGQK	C_2_H_2_	1	IIb	2	XP_007046206.1
Tc04_g029800	WRKYGQK	C_2_H_2_	1	IIc	4	XP_007035842.1
Tc01_g010370	WRKYGQK	C_2_H_2_	1	IIc	1	XP_007048165.1
Tc06_g013130	WRKYGQK	C_2_H_2_	1	IIc	6	XP_007025165.1
Tc01_g039500	WRKYGQK	C_2_H_2_	1	IIc	1	XP_007052352.1
Tc01_g014750	WRKYGQK	C_2_H_2_	1	IIc	1	XP_007048873.1
Tc00_g047270	WRKYGQK	C_2_H_2_	1	IIc	0	XP_007043929.1
Tc04_g004210	WRKYGQK	C_2_H_2_	1	IIc	4	XP_007031915.1
Tc01_g031960	WRKYGQK	C_2_H_2_	1	IIc	1	XP_007051149.1
Tc05_g004380	WRKYGQK	C_2_H_2_	1	IIc	5	XP_007026988.1
Tc00_g076580	**WRKYGKK**	C_2_H_2_	1	IIc	0	XP_007037699.1
Tc01_g035290	WRKYGQK	C_2_H_2_	1	IIc	1	XP_007051697.1
Tc08_g013540	**WRKYGKK**	C_2_H_2_	1	IIc	8	XP_007019683.1
Tc03_g015140	WRKYGQK	C_2_H_2_	1	IIc	3	XP_007038026.1
Tc01_g010220	**WRKYGKK**	C_2_H_2_	1	IIc	1	XP_007048143.1
Tc00_g017270	**WRKYGKK**	C_2_H_2_	1	IIc	0	XP_007037701.1
Tc09_g005290	**WRKYGKK**	C_2_H_2_	1	IIc	9	XP_007012369.1
Tc02_g032350	WRKYGQK	C_2_H_2_	1	IIc	2	XP_007045916.1
Tc05_g027100	WRKYGQK	C_2_H_2_	1	IId	5	XP_007030538.1
Tc09_g000780	WRKYGQK	C_2_H_2_	1	IId	9	XP_007011614.1
Tc01_g005580	WRKYGQK	C_2_H_2_	1	IId	1	XP_007047364.1
Tc03_g025390	WRKYGQK	C_2_H_2_	1	IId	3	XP_007039903.1
Tc01_g027130	WRKYGQK	C_2_H_2_	1	IId	1	XP_007050397.1
Tc08_g000030	WRKYGQK	C_2_H_2_	1	IId	8	XP_007017155.1
Tc03_g028030	WRKYGQK	C_2_H_2_	1	IIe	3	XP_007040356.1
Tc06_g000970	WRKYGQK	C_2_H_2_	1	IIe	6	XP_007022995.1
Tc03_g019750	WRKYGQK	C_2_H_2_	1	IIe	3	XP_007099721.1
Tc06_g013990	WRKYGQK	C_2_H_2_	1	IIe	6	XP_007025322.1
Tc01_g035330	WRKYGQK	C_2_H_2_	1	IIe	1	XP_007051705.1
Tc01_g031780	WRKYGQK	C_2_H_2_	1	IIe	1	XP_007051114.1
Tc10_g016560	WRKYGQK	C_2_HC	1	III	10	XP_007011366.1
Tc03_g017550	WRKYGQK	C_2_HC	1	III	3	XP_007038956.1
Tc03_g028700	WRKYGQK	C_2_HC	1	III	3	XP_007040478.1
Tc01_g034680	WRKYGQK	C_2_HC	1	III	1	XP_007051596.1
Tc04_g016130	WRKYGQK	C_2_HC	1	III	4	XP_007033512.1
Tc10_g016570	WRKYGQK	C_2_HC	1	III	10	XP_007011367.1
Tc02_g001230	**WRKHGQT**	C_2_HC	1	III	2	XP_007041160.1
Tc02_g001200	WRKYGQK	C_2_HC	1	III	2	XP_007041155.1
Tc00_g017240^a^*	**WRCIGIK**	C_1_H_2_	1	nd	0	XP_007037703.1
Tc02_g001170^a^*	**RTKYYRC**	C_1_HC	1	nd	2	XP_007041153.1
Tc02_g012180^a^**	No conserved stretch	C_2_H_2_^b^	1	nd	2	XP_007043029.1

^a^ Sequences not classified in any group due to the absence of WRKY domain, incomplete zinc finger motif and/or small conserved WRKY domain.

^b^ Zinc finger C_2_H_2_ identified by the Smart program (http://smart.embl-heidelberg.de/)

* Excluded from the phylogenetic analyses.

** Excluded from all the subsequent analyses.

### *TcWRKY* gene classification and chromosomal location

After confirmation and identification of the final dataset of TcWRKY proteins (S1 Fig), all whole protein sequences, except the Tc02_g012180, were used for sequence alignment using the ClustalOMEGA software (http://www.ebi.ac.uk/Tools/msa/clustalo/). The cacao TcWRKY proteins were categorized based on Arabidopsis WRKY protein classification [6]. The distribution of the *TcWRKY* sequences on cacao chromosomes was obtained from the CacaoGenDB database v1.0 (http://cocoagendb.cirad.fr) [28] using “WRKY” as input in the “Search by Keywords” tool (http://cocoa-genome-hub.southgreen.fr/content/search-keywords). The sequences downloaded from the CacaoGenDB database v1.0, excluding the chromosome Tc00, were used as an input file for prediction of *WRKY* gene duplication and collinearity using the MCScanX toolkit, according to the manual [29]. This analysis was made independently from other previously published reports [28] mainly because the CacaoGenDB database was regularly updated since the database creation and work publication.

### Identification of conserved motifs in TcWRKY proteins

The detection of the motif composition in the 58 identified cacao WRKY proteins (all proteins except Tc00_g017240, Tc02_g001170 and Tc02_g012180) was performed with the MEME 4.9.1 program (http://meme.nbcr.net/meme/intro.html) [30]. MEME represents motifs as position-dependent letter-probability matrices which describe the probability of each possible letter at each position in the pattern [30]. The maximum number of motifs was set at 20, the maximum motif length was set at 80 amino acids, the optimum motif width was constrained to be between 6 and 300 residues, and the other parameters were used with the default settings. The organization of the different motifs (e.g. WRKY DNA-binding domain, leucine rich repeat/LRR) present in the cacao WRKY proteins was performed with the SuperFamily database v.1.75 (http://supfam.org/SUPERFAMILY/; [31]) based on hidden Markov models.

### Phylogenetic analysis

A phylogenetic tree of amino acid sequences of WRKY domains from cacao (58 sequences, see paragraph above) and *Arabidopsis* (S1 Table) was constructed. The amino acid sequences of WRKY domains were aligned using the MUSCLE program v3.6 [32] with default parameters. The MEGA 5.1 software was used to construct a rooted phylogenetic tree [33]. The tree based on WRKY domains of *Arabidopsis* and cacao was used to identify and classify putative orthologs. The statistical method used to construct the tree was neighbor-joining [34], the evolutionary distances were obtained using the p-distances method, and these distances were used to estimate the number of amino acid substitutions per site. The reliability of each tree was established by conducting 1000 bootstrap sampling steps. To construct the tree with all species used in this study, the JTT evolutionary model plus gamma-distributed rate (JTT+G) was used as determined by the Modeltest program version 3.7 [35]. The phylogenetic analysis was completed by an analysis of orthology using the plant transcription factor database v.3.0 (http://plntfdb.bio.uni-potsdam.de/v3.0).

### Plant material

The plant material used in this study consisted in TSH1188 and Catongo *T*. *cacao* genotypes. TSH1188 was chosen based on its demonstrated resistance to witches’ broom disease from field progeny trials assessed by the number of vegetative and cushion brooms per plant and per year, and by witches’ broom incidence scale [36, 37]. The susceptible cultivar Catongo was chosen as the standard for susceptibility. These genotypes were previously used as resistance and susceptibility standards in several molecular and histological studies of witches’ broom disease [38–42]. Seedlings, derived from open-pollinated pods of both genotypes were planted in a mixture of commercial potting mix (Plantmax^®^, Eucatex, São Paulo, SP, Brazil) and clay-rich soil, in a 2:1 proportion, and grown in sterile substrate in the greenhouses of CEPLAC/CEPEC (Bahia, Brazil) under natural light and 90% relative humidity until the inoculation day. All the experiments followed a complete randomized design.

### Plant inoculation procedure

The inoculation procedure was conducted in the greenhouses of CEPLAC/CEPEC (Bahia, Brazil) using inoculum from *Moniliophthora perniciosa* isolate 4145, which has been maintained in the CEPLAC/CEPEC phytopathological *M*. *perniciosa* collection (CEGEN n°109/2013/SECEXCGEN) in sterile distilled water [43] and in mineral oil. The inoculation procedure has been previously described in detail [42]. Briefly, apical shoot apexes of 300 4-week-old seedlings from each genotype were inoculated with a 20 μl drop of a basidiospore suspension (2.10^5^ basidiospores ml^-1^ with >80% germination) in 0.3% agar [44]. Inoculated seedlings were incubated for 48 h in a control dark moist chamber at 23±2°C and relative humidity greater than 97%. Afterwards seedlings were transferred to an acclimatization greenhouse with 23±2°C temperature and irrigation for 20 minutes three times a day until the end of the experiment. The greenhouse relative humidity was around 80% controlled through an automated fogging system. Seedlings mock-inoculated with sterile 0.3% were used as controls. Symptoms were observed weekly up to 60 days after inoculation (dai). The inoculation efficiency was checked based on the inoculum viability (>80% of spore germination observed 24 h after inoculation [hai]) and on disease incidence on the susceptible cultivar Catongo (>80% of disease incidence observed 60 dai). Apical shoot apexes were harvested at 6, 12, 24, 48 and 72 hai and 7, 15, 30 and 45 dai. These harvesting points correspond to the main disease stages as previously described [40–42, 45, 46]. Briefly, 6h corresponded to *M*. *perniciosa* penetration in resistant and susceptible genotypes. The 24 to 72 hai period corresponded to the early stages of the infection; at 48 h the fungus hyphae were observed in the cortex beneath the epidermal layer in the susceptible genotype (in the resistant genotype the infection and fungus progression were reduced or stopped). From 15 to 25 dai, macroscopically the symptoms on susceptible genotype were observed as apical swellings and slight morphological alterations of the shoots (S2 Fig), and microscopically, hyphal strands were seen in the cortex and grew toward the vascular bundles through xylem ray cells. At 30 dai, apical hypertrophy and swellings were observed (S2 Fig); at this time the pathogen reached the pith, and swollen, flexuous, septated and unclamped hyphae grew in the apoplast. The infection continued to develop forming the phenotypic response of terminal green brooms that could be seen at 45 dai (S2 Fig). At 60 dai, the infected plant presented macroscopic symptoms called dry broom (data not shown). Control plants were kept and harvested under the same conditions and at the same time points. For each genotype and at each harvesting time (for inoculated and control plants), 24 samples were collected (1 sample = 1 apical apex of 1 cacao plantlet) and immediately frozen in liquid nitrogen and stored at -80°C until use. Then three samples collected from each genotype at each harvesting time were pooled forming one biological replicate; two biological replicates were obtained (i.e., 6 apexes from the 24 collected were used).

### Total RNA extraction and cDNA synthesis

Cacao samples were macerated in liquid nitrogen until a fine powder was obtained. Total RNA was extracted from 100–150 mg of macerated tissue using the RNAqueous® Total RNA isolation kit according to the manufacturer’s instructions (Thermo Scientific) with modifications as previously described [47]. Briefly, after the addition of the lysis buffer to the macerated samples, a sonication step was added (10 s pulse/min, 70% output; Gex Ultrasonic processor 130, 130 W) to break polysaccharides which are present in high levels in cacao tissues. This step was conducted on ice. RNA was quantified using a NanoDrop 2000 spectrophotometer (Thermo Scientific) and its integrity was checked by 1% agarose gel electrophoresis. RNA was treated by DNAse I RNase-free according to the manufacturer’s instructions (Invitrogen). The cDNA was synthesized from 200 ng of RNA using the RevertAid First Strand cDNA kit according to the manufacturer’s instruction (Thermo Scientific). The cDNA quantification was carried out in the same NanoDrop 2000 spectrophotometer.

### Primer design and qPCR analysis

Seven cacao *WRKY* genes (Tc04_t016130, Tc10_t016570, Tc09_t001530, Tc06_t004420, Tc06_t013130, Tc01_t014750, Tc01_t018460) were selected based on the phylogenetic analysis and searches for genes that are well characterized and possibly involved in plant defense mechanisms against pathogenic fungi in the Arabidopsis genus. Specific primers were designed for each gene using the OligoPerfect™ Designer tool (http://tools.thermofisher.com) according to the following criteria: i) amplicon size of 65–150 bp; ii) primer length of 17–23 bases; iii) melting temperature of 57–63°C; and iv) GC content of 40%-80% (S2 Table and S3 Table). The OligoAnalyzer v.3.1 program (https://www.idtdna.com/calc/analyzer) was used to analyze the primer pairs in relation to hairpin loop, self-dimer and hetero-dimer formation (https://www.idtdna.com/calc/analyzer). Primers were also designed to amplify specific regions presenting different sizes, melting temperatures, GC contents and GC/AT ratios (S3 Table) to avoid cross-reaction between genes from the cacao WRKY family [48]. For qPCR analysis, two reference genes (malate dehydrogenase/MDH and glyceraldehyde 3-phosphate dehydrogenase/GAPDH) previously described in cacao meristems infected by *M*. *perniciosa* [47, 49] were used (S2 Table). Expression analysis by qPCR was conducted in an Agilent Technologies Stratagene Mx3005P system (Agilent Technologies). The qPCR reaction consisted of 200 ng of cDNA, 0.5 μM of each primer from candidate or reference genes (S2 Table) and 1X of Maxima^**™**^ SYBR Green/ROX qPCR Master Mix (Thermo Scientific) in a total volume of 12.5 μl. Cycling conditions were: 50°C for 2 min, 95°C for 1 min followed by 40 cycles at 95°C for 30 s, 58°C for 45 s and 72°C for 30 s, with detection of the fluorescent signal at the end of each extension cycle. To verify that each primer pair produced only a single PCR product, dissociation analysis was carried out under the following cycling conditions: 95°C for 25 s, 58°C for 30 s and 72°C for 30 s. The amplification efficiency of each primer pair was analyzed using three amounts (50, 100 and 200 ng) of each cDNA sample. Experiments also included a negative control (no template DNA). Real-time data acquisition was performed with the Stratagene MX3005P system containing the MxPro QPCR software (Agilent Technologies), which provided the values of cycle threshold (Ct) and of fluorescence. Amplification efficiency (E) was accessed using the Miner 2.2 software [50]. The gene expression level was analyzed with three experimental repetitions for both Catongo and TSH1188 genotypes with the comparative Ct method (2^**-ΔΔCt**^) using: i) MDH and GAPDH as reference genes (average of expression values from both genes); and ii) non-inoculated plants as a calibrator (at each harvesting time, a non-inoculated sample was collected and used as calibrator of the corresponding inoculated sample). Statistical analysis was done using the SASM-Agri software v.8.2 [51], which tested the experiments as a completely randomized design. *t*-test and *F*-test (ANOVA) were applied with a critical value of 0.05. The Duncan test (*P* ≤ 0.05) was employed for mean separation when *F*-values were significant.

## Results

### Identification and classification of TcWRKY sequences

**Fig 1:**
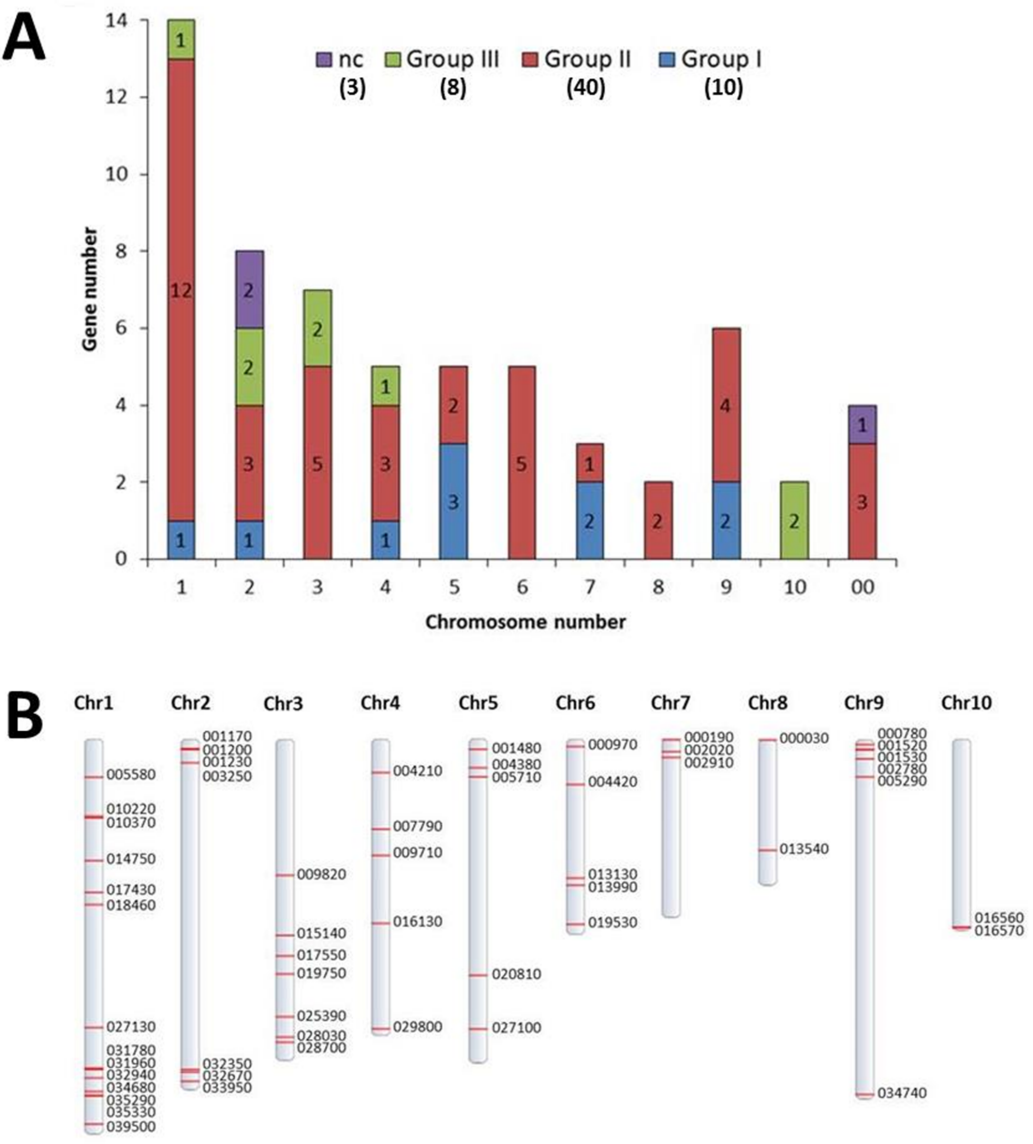
Classification of *TcWRKY* genes and distribution on cacao chromosomes. **A.** Distribution of *TcWRKY* gene groups among cacao chromosomes. Chromosome 00 corresponds to non-anchored genome regions. nc: not classified. The total number of genes in each group was indicated under parenthesis. **B.** Physical distribution of the *TcWRKY* genes on cacao chromosomes. In CocoaGenDB, the sequence names are preceded by “Tc_g” (e.g., Tc_g005580).

**Fig 2:**
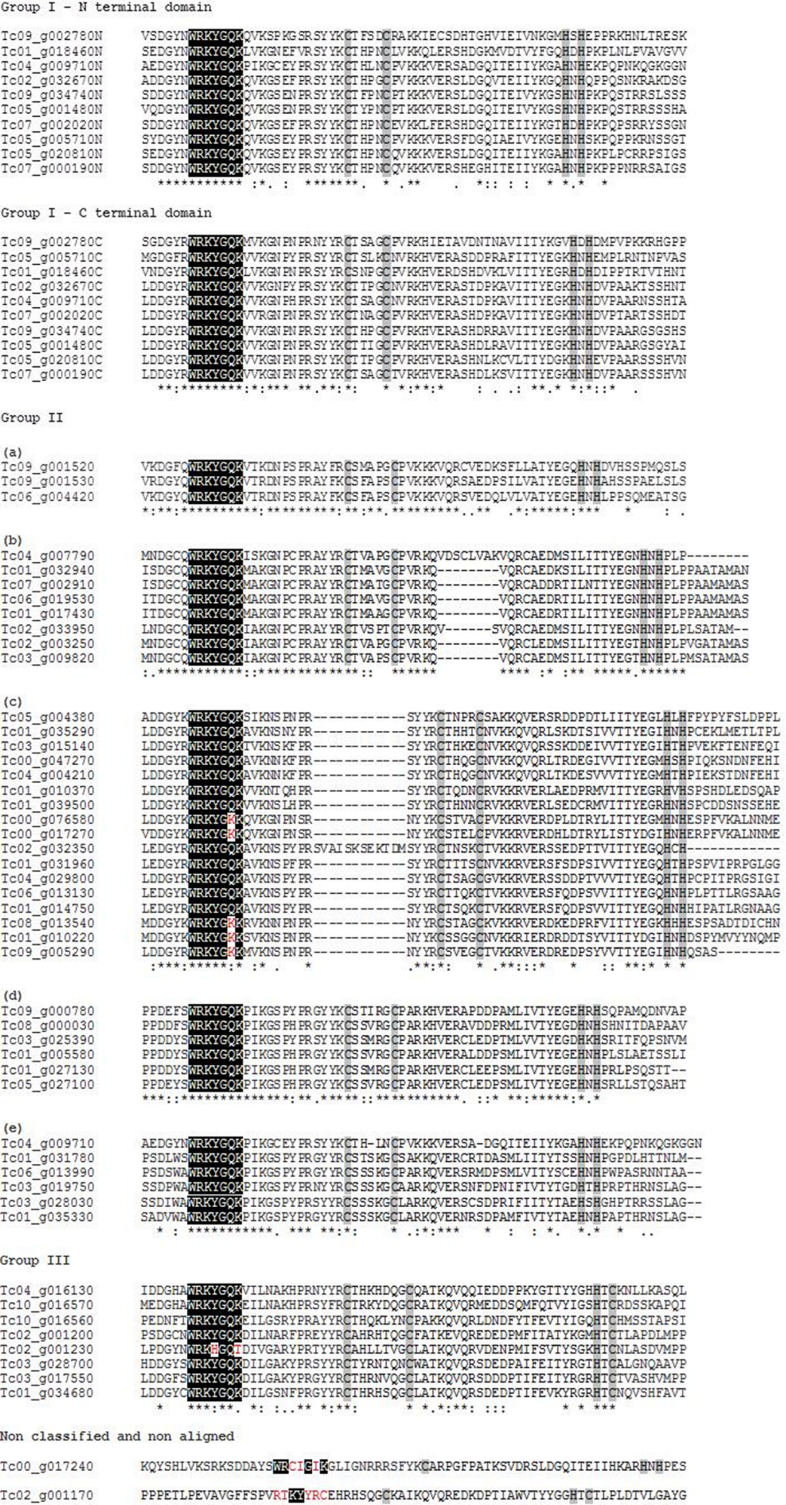
Multiple alignments of 60 TcWRKY proteins. The whole TcWRKY protein sequences were aligned but only the conserved domains are presented. The conserved WRKY heptapeptide is indicated in black; variations of the heptapeptide are indicated in red. The zinc finger domain is indicated in grey. Gaps introduced to get the best alignment are indicated by (-). Within each group, (*) represents identical amino acids, (.) and (:) represent conserved substitutions and semi-conserved substitutions, respectively.

In the cacao genome, 61 non-redundant sequences corresponding to putative WRKY proteins were found using BLASTP (Table 1). Among them, 47 had been previously annotated as WRKY proteins in the CocoaGenDB; the other 14 proteins identified by BLASTP had been previously annotated as uncharacterized, predicted or hypothetical proteins (data not shown). Among the 61 non redundant sequences, 60 contained at least one complete heptapeptide WRKY motif while one sequence (Tc02_g012180) did not present such a conserved stretch (Table 1). The TcWRKY proteins were classified into three groups according to the presence of WRKY motif and the zinc-finger motif type. Group I contained two WRKY motifs (one in the N-terminal region of the sequence, the other in the C-terminal region) and two C_2_H_2_ zinc-finger motifs; this group contained 10 TcWRKY proteins (Table 1; Fig 1A). Group II contained only one WRKY motif and a C_2_H_2_ zinc-finger motif (40 TcWRKY proteins). Group III contained only one WRKY motif and a C_2_HC zinc-finger motif (8 TcWRKY proteins) (Table 1, Fig 1A). It was not possible to classify the three other putative WRKY proteins (Tc02_g017240, Tc02_g001170 and Tc02_g012180) because of the presence of a highly altered WRKY motif and/or zinc-finger motif, or because of the absence of a WRKY motif (Table 1; Fig 1A). The zinc finger motifs C_2_H_2_ from the group I were CX_4_CX_22_HX_1_H (N-terminal) and CX_4_CX_23_HX_1_H (C-terminal) for all sequences (Fig 2). Group II was divided into five subgroups (IIa to e) according to C_2_H_2_ zinc-finger structure. Subgroups IIa, IIb, IIc, IId and IIe were found to contain 3, 8, 17, 6 and 6 genes, respectively (Table 1) and the members of subgroups IIa, IId, IIe and 6 members of subgroup IIb showed the CX_5_CX_23_HX_1_H zinc-finger motif. The other two members of subgroup IIb (Tc02_g033950 and Tc04_g007790) showed the CX_5_CX_25_HX_1_H and CX_5_CX_31_HX_1_H zinc-finger structures, respectively (Fig 2). All the members of group IIc showed the CX_4_CX_23_HX_1_H zinc-finger structure (Fig 2). In the case of group III, the zing-finger motif was CX_7_CX_23_HX_1_C (Fig 2). The WRKY domain (WD) was highly conserved in 52 proteins, but some of them presented variations (Table 1; Fig 2). The proteins of the group IIc showed a WRKY motif with only one amino acid modification; the protein Tc02_g001230 showed a WRKY motif with two amino acid modifications (WRKHGQT) while Tc00_g017240 contained a WRKY motif with three modifications (WRCIGIK) in addition to the presence of an incomplete zinc-finger motif (Table 1, Fig 2). These seven proteins belong to subgroup IIc, III, or were non-classified (Table 1). The sequence Tc02_g012180, which did not contain any WRKY motif, was removed from all subsequent analyses, while the sequences Tc02_g001170 and Tc02_g012180, which showed modified WRKY motif and modified zinc-finger motif, were excluded from phylogenetic analysis.

### Distribution of *WRKY* genes in the cacao genome

**Fig 3:**
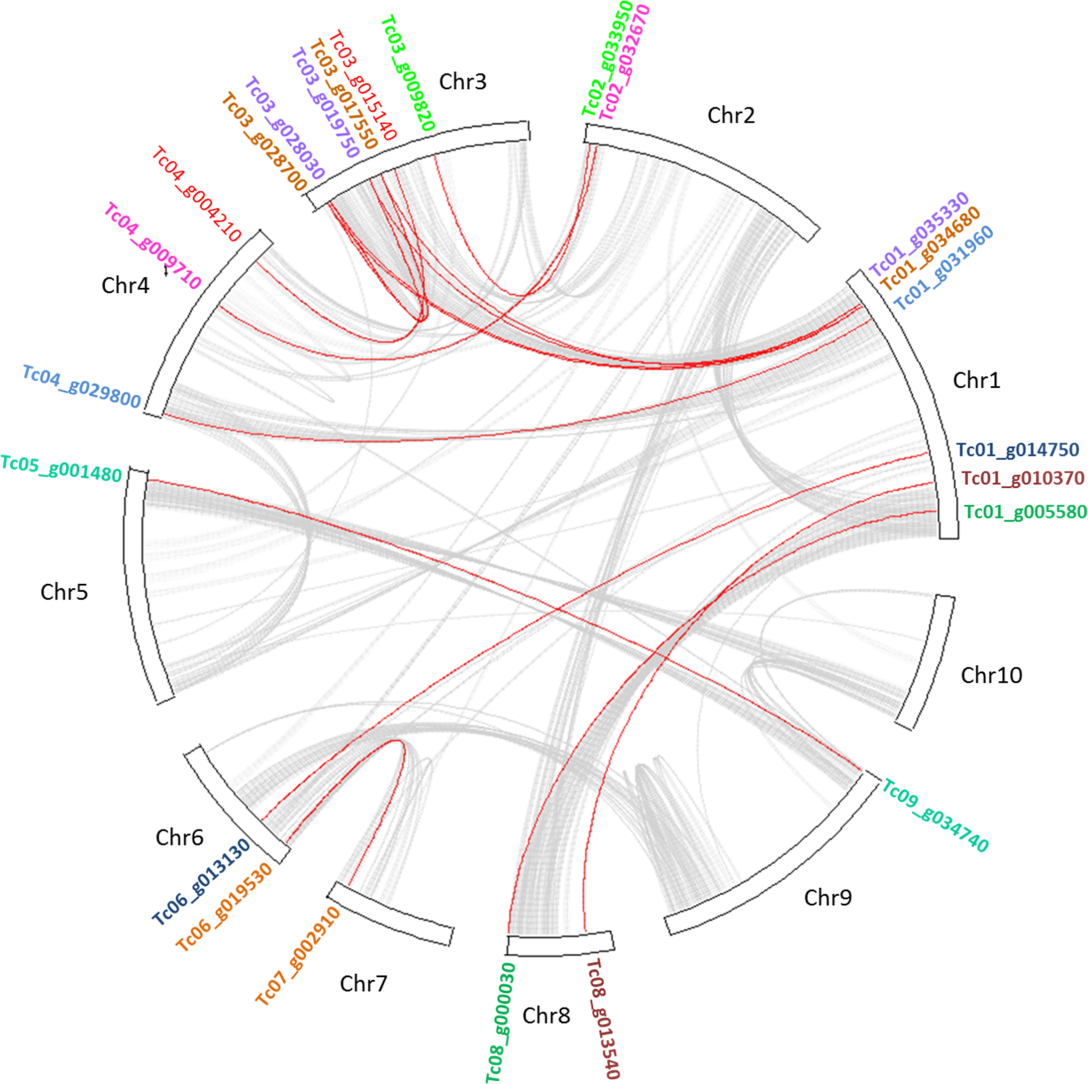
Schematic representation of interchromosomal relationships of *TcWRKY* genes. Gray lines indicate all syntenic blocks in the cacao genome, whereas the red lines suggest duplicated *WRKY* gene pairs. The corresponding *WRKY* gene names are indicated, duplicated genes are marked with the same color. The chromosome number is indicated at the top of each chromosome.

The 61 putative *WRKY* sequences were distributed on the 10 cacao chromosomes (Fig 1A and 1B). A higher abundance of *WRKY* genes was observed on chromosome 1: 14 genes belonging to groups I (1 gene), II (12) and III (1) (Fig 1A and 1B). In contrast, chromosomes 8 and 10 contained only two *WRKY* genes each (from groups II and III, respectively; Fig 1A). The other *WRKY* genes were distributed as follows: 8, 7, 5, 5, 5, 3 and 6 on chromosomes 2, 3, 4, 5, 6, 7 and 9, respectively, and belonged mainly to groups I and II (Fig 1A). For four of the genes (Tc00_g047270, Tc00_g076580, Tc00_017270, Tc00_g017240), the location was uncertain, so the genes were distributed on “chromosome 00”, corresponding to non-anchored sequences of the genome (Table 1; Fig 1A). Because tandem and segmental duplication play an important role in the expansion of multigene families, we analyzed the syntenic regions and structural changes of all 10 cacao chromosomes (Fig 3). Twenty-four *WRKY* genes were identified in segmental duplication events in the cacao genome (Fig 3). Tc01_g035330 participated in two duplication events with Tc03_g019750 and Tc03_g028030, while Tc01_g034680 also participated in two duplication events with Tc03_g017550 and Tc03_g028700. *TcWRKY* genes were located within syntenic blocks of all chromosomes except chromosome 10. Most of the duplications were located in chromosomes 1 and 3 (Fig 3).

### Phylogenetic analysis of TcWRKY proteins

**Fig 4:**
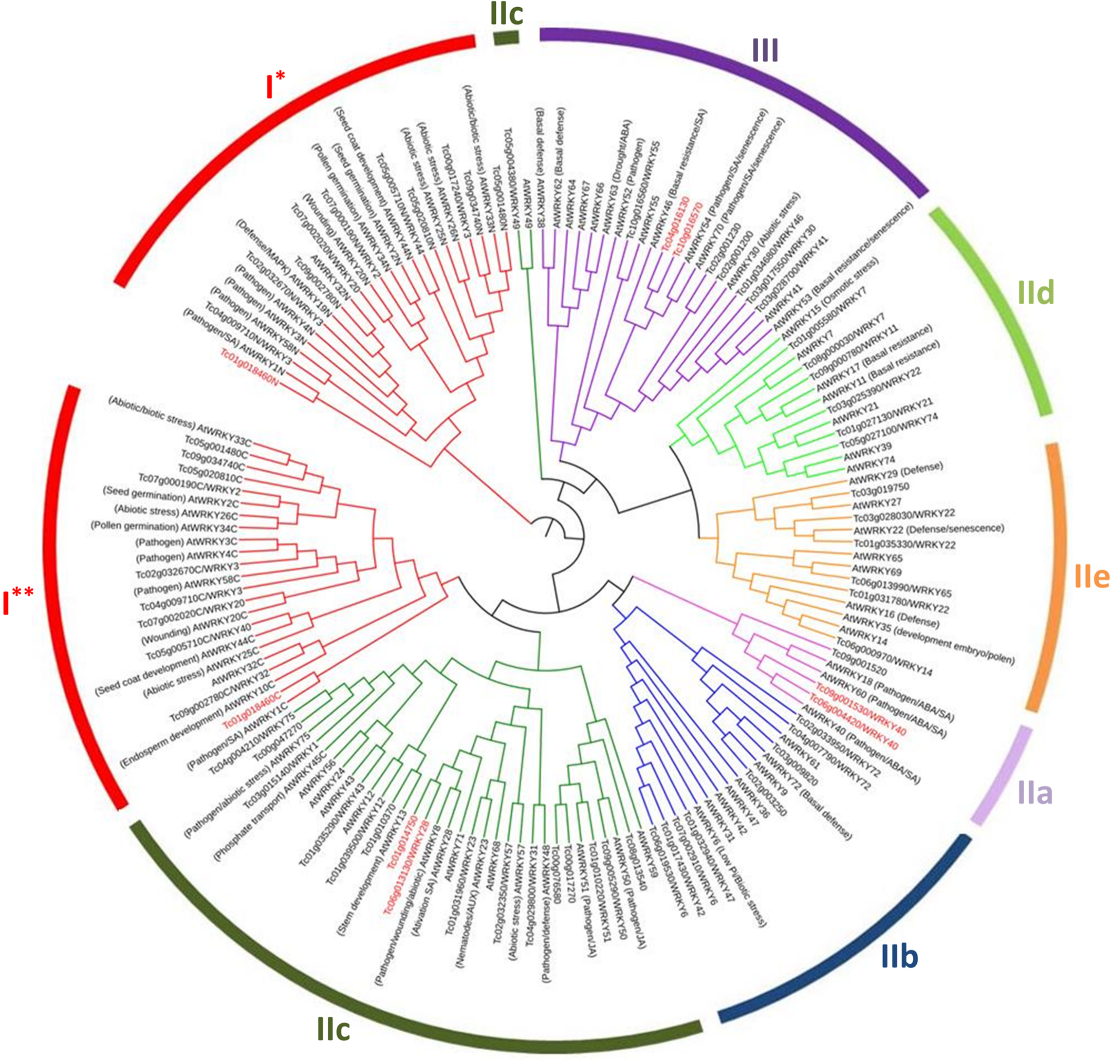
Phylogenetic tree of WRKY protein domains from cacao and Arabidopsis. TcWRKY protein domains (S1 Table) were grouped into three groups and their subgroups as follows: group I in red, subgroup IIa in light purple, subgroup IIb in blue, subgroup IIc in dark green, subgroup IId in light green, subgroup IIe in orange, group III in dark purple. (*) and (**) indicate the N-terminal and C-terminal WD from group I genes. Cacao WRKY proteins possibly involved in plant defense response and selected for gene expression analysis are indicated in red. These proteins are: Tc04_g016130, Tc10_g016570, Tc09_g001530, Tc06_t004420, Tc06_t013130, Tc01_t014750 and Tc01_t018460 (appearing in the I* and I** groups).

A phylogenetic tree of WRKY amino acid domains from cacao and *Arabidopsis* (S1 Table) was constructed to investigate the relationship between these two species (Fig 4). The WRKY protein domains were grouped according to the general WRKY classification (group I, IIa-e, III). The group I was subdivided according to the N-terminal and C-terminal WD (I* and I**, respectively). The least consistent group was the IIc that was split in two branches, one more related to the group III, the other to the group I**. Groups IIa and IIb presented a close phylogenetic relation, as well as IId and IIe (Fig 4). The phylogenetic tree constructed with cacao and *Arabidopsis* WRKY domains also allowed inferences to be made about the possible function of cacao sequences based on *Arabidopsis* sequence function knowledge. Clades or sequences associated with plant responses to pathogen or defense inducers (such as abscisic acid or salicylic acid) were identified and cacao sequences with possible defense-related responses were selected for expression analysis (Fig 4). Similar results were obtained by analysis of orthology between cacao and *A*. *thaliana*: Tc01_g018460 (XP_007049282.1) was orthologue to AT2G04880.2 (WRKY3); Tc04_g016130 (XP_007033512.1) to AT3G56400.1 (WRKY70); Tc10_g016570 (XP_007011367.1) to AT3G56400.1 (WRKY70); Tc09_g001530 (XP_007011727.1) to AT4G31800.2 (WRKY18); Tc06_g004420 (XP_007023430.1) to AT1G80840.1 (WRKY40); Tc06_g013130 (XP_007025165.1) to AT4G18170.1 (WRKY28); and Tc01_g014750 (XP_007048873.1) to AT1G29860.1 (WRKY28).

**Fig 5:**
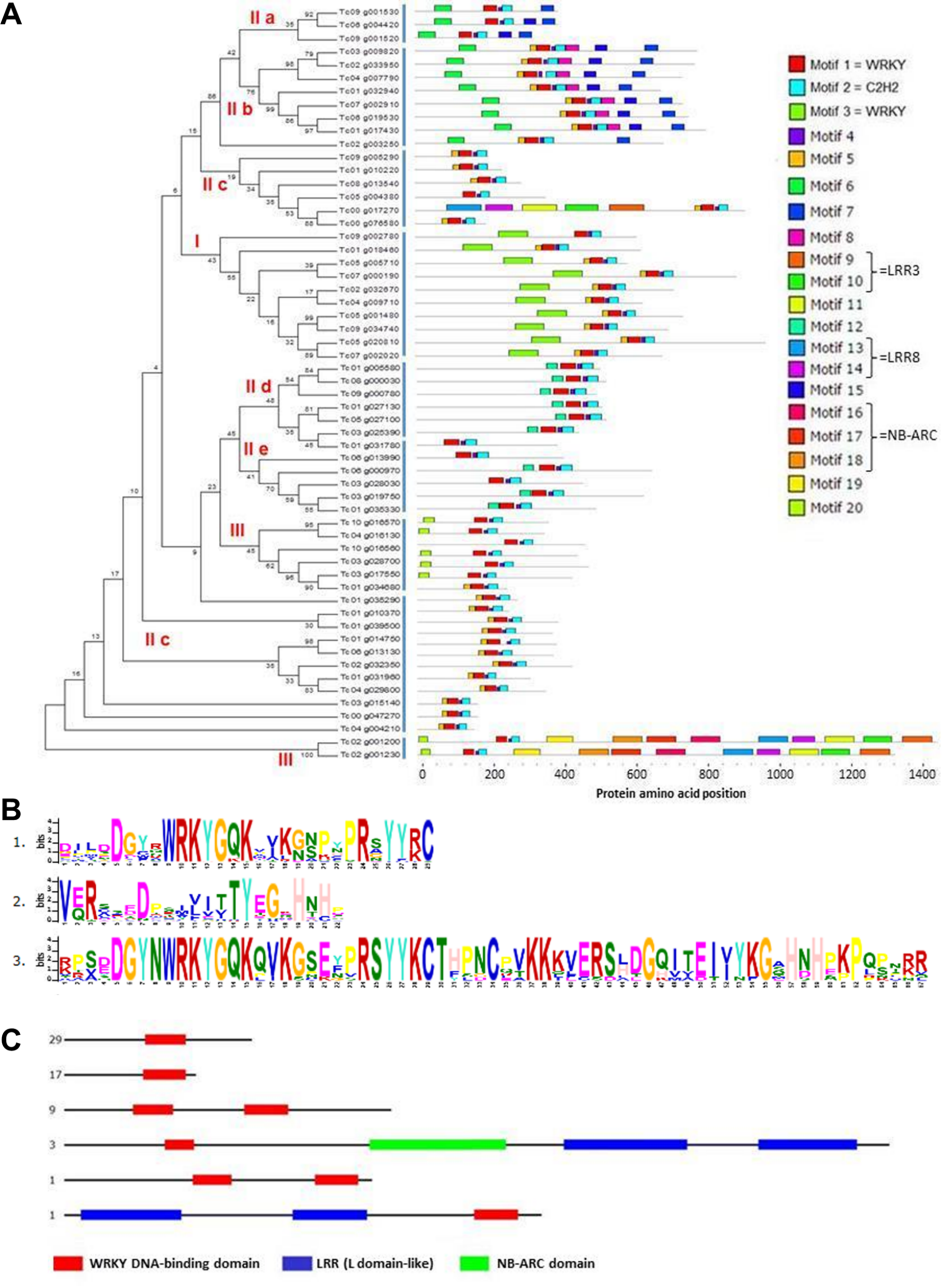
Phylogenetic tree and motif composition of TcWRKY proteins. **A.** Phylogenetic tree (left side of the figure) and motif composition of the TcWRKY proteins (right side), obtained using the MEGA v.5.1 and the MEME programs, respectively. The phylogenetic tree contains 58 TcWRKY proteins (excluding Tc00_g017240, Tc02_g001170 and Tc02_g012180). WRKY groups are indicated in red. **B.** Motif detail of the three first most probable motifs (WRKY C-terminal, C_2_H_2_ and WRKY N-terminal motifs) of the TcWRKY proteins, obtained by the MEME program. **C.** Organization of the 60 TcWRKY proteins (excluding Tc02_g012180) in 6 categories by domain combination using the SuperFamily database. The numbers indicated on the left represent the number of cacao WRKY proteins in each category.

To investigate the relationship between WRKY family members in cacao more thoroughly, we analyzed the motif pattern of the TcWRKY sequences (Fig 5A). The different motifs were identified based on the biochemical properties of their amino acids as well as their specific location in the protein sequence [52]. The conserved amino acids, the position of each residue in the WRKY sequence, as well as the residue that varied according to the protein sequence are presented in Fig 5A. Twenty motifs were found and 3 of them (motifs 1 to 3; Fig 5B) constituted the WD. Motifs 1 and 2, corresponding to the C-terminal WRKY and the C_2_H_2_ motifs, were present in 58 TcWRKY members. Motif 3 corresponded to the N-terminal WRKY motif (10 members). Motif 4 is an intermediary amino acid region between motifs 1 and 2, forming the complete WD with approximately 60 amino acids. This motif is present in 58 TcWRKY proteins (Fig 5A and 5B). Six different combinations containing the WRKY motifs were found in the proteins identified in the cacao genome (Fig 5C). Twenty-nine presented the WRKY motif in the center of the sequence and 18 in the C-terminal region. Of the 10 members of group I (with duplicated domains), 9 members presented the WRKY motif in the central region of the protein while one presented this domain in the C-terminal region. Three other members (Tc02_g001230, Tc02_g001200 e Tc02_g001170) presented the WRKY motif domain in the N-terminal region of the protein and also contained the 2 LRR motifs and one NB-ARC domain. One sequence, Tc05_g005710, contained 2 LRR motifs (Fig 5C).

### Expression patterns of seven selected *TcWRKY* genes in resistant and susceptible *Theobroma cacao* genotypes

**Fig 6:**
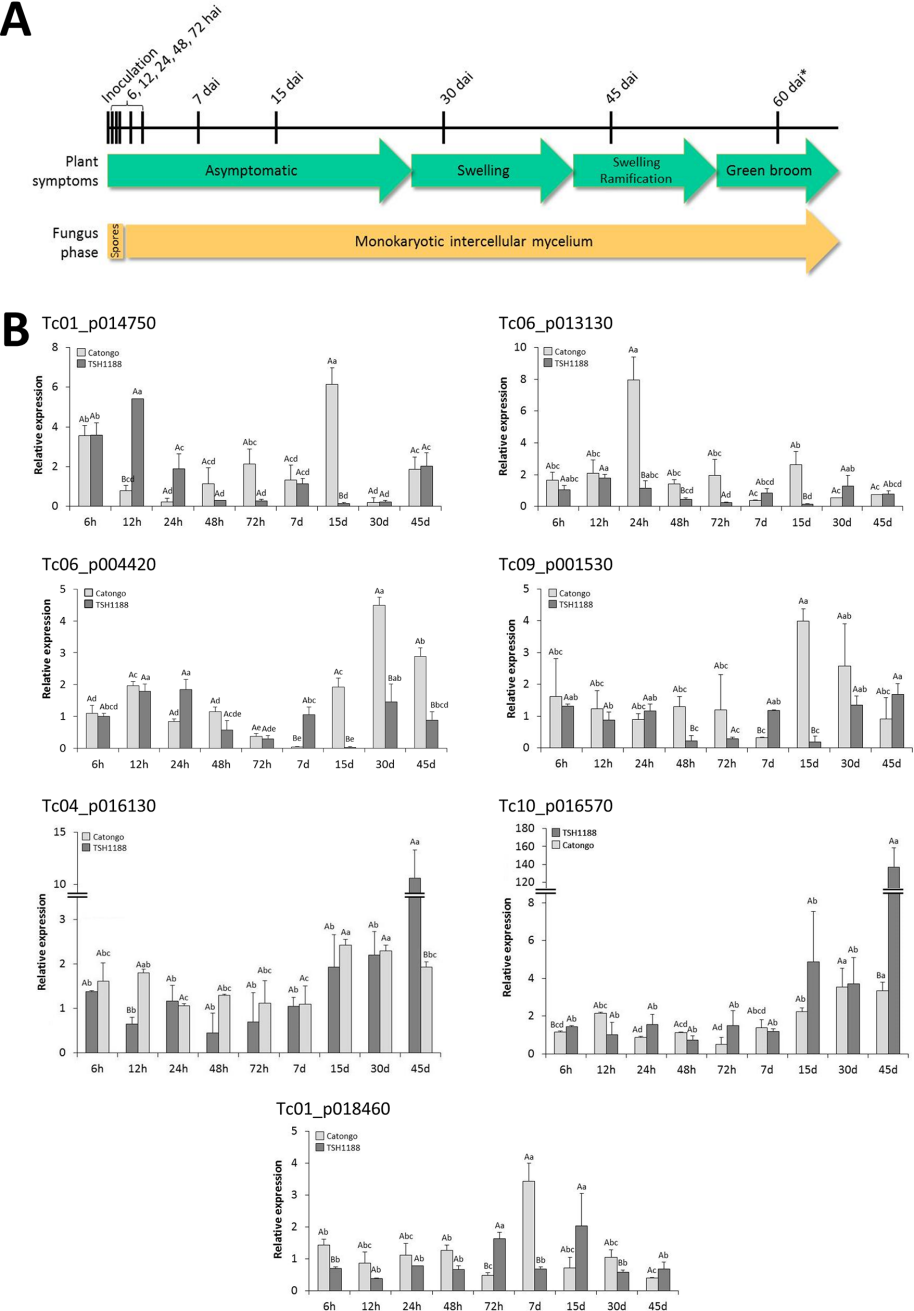
Expression profile of seven *TcWRKY* genes in resistant (TSH1188) and susceptible (Catongo) cacao plants inoculated with *M*. *perniciosa*. **A.** Representation of the plant symptoms and fungus phase during the infection time course in Catongo genotype. The harvesting times of inoculated and control plants are indicated on the top of the figure, excepted (*) that was used only of plant symptoms observation. **B.** RT-qPCR of *TcWRKY* genes. The control used as calibrator in the expression value calculation corresponds to the control plants (mock-inoculated with water) collected at each harvesting time and used as calibrators of the corresponding inoculated sample (see also Methods section). The results are the arithmetical mean of the repetitions ± standard error. Different lower case letters indicate significant statistical difference between harvesting times for each genotype by the Duncan test (*P* ≤ 0.05), while upper case letters correspond to significant statistical difference between genotypes for each harvesting time by *t*-test (*P* ≤ 0.05). d: days after inoculation; h: hours after inoculation.

According to the phylogenetic analysis (Fig 4), the orthology between cacao and *Arabidopsis* and the putative function of *WRKY* genes in *Arabidopsis* (related to response to biotic stress), 7 *TcWRKY* (Tc04_g016130, Tc10_g016570, Tc09_g001530, Tc06_t004420, Tc06_t013130, Tc01_t014750 and Tc01_t018460) genes were selected for expression analysis by RT-qPCR. From the 7 *TcWRKY* genes analyzed, one belonged to group I (Tc01_t018460), two to subgroup IIa (Tc06_t004420, Tc09_g001530), two to subgroup IIc (Tc01_t014750, Tc06_t013130) and two to group III (Tc04_g016130, Tc10_g016570). The expression of the *TcWRKY* genes was analyzed in two cacao genotypes, TSH1188 (resistant to witches’ broom disease) and Catongo (susceptible) infected or not (control) with *M*. *perniciosa* (Fig 6A and 6B). For both genotypes and for all the harvesting points, the PCR amplification occurred at the same melting temperature, showing that only the target gene was amplified (data not shown). RT-qPCR analysis showed differential expression between genotypes and/or between time intervals for all the analyzed genes. In Catongo, the *TcWRKY* genes Tc06_p004420, Tc09_p001530, Tc04_p016130 and Tc10_p016570 showed higher transcript abundance in the final stages of the infection (15 to 45 dai). The gene Tc01_p014750 showed higher transcript abundance 15 dai but also at the beginning of the infection (6 hai; about 6 times more expressed). The gene Tc06_p013130 showed higher transcript abundance (about 7 times more than the control) 24 hai while the gene Tc01_p018460 was more expressed 7 dai (Fig 6B). In TSH1188, the transcript abundance of the genes Tc06_p013130, Tc06_p004420, Tc09_p001530 and Tc01_p018460 was low (about 2 times more expressed than control). The gene Tc01_p014750 was mainly expressed in the initial infection points (6 to 24 hai; about 5 times more expressed). Interestingly, the Tc04_p016130 and Tc10_p016570 *TcWRKY* genes showed very high transcript abundance 45 dai: Tc04_p016130 showed an increase of about 12 times while Tc10_p016570 was expressed 120 times more than the control (Fig 6B). Some significant differences were also observed between genotypes: 12 hai and 15 dai for the gene Tc01_p014750; 24 hai, 48 hai and 15 dai for the gene Tc06_p013130; 7, 15, 30 and 45 dai for the gene Tc06_p004420; 48 hai, 7 and 15 dai for the gene Tc09_p001530; 12 hai and 45 dai for the gene Tc04_p016130; 6 hai and 45 dai for the gene Tc10_p016570; and 6 and 72 hai, 7 and 30 dai for the gene Tc01_p018460 (Fig 6B).

## Discussion

WRKY proteins constitute one of the most important transcription factor families in plants due to their participation in diverse biological processes, including responses to biotic and abiotic stresses [7]. A better understanding of this family, including member characterization, phylogenetic analysis and expression analysis, can help to define new disease management strategies, as in the case of the cacao-*M*. *perniciosa* interaction. In this study, based on sequence comparison and molecular phylogeny, 58 cacao proteins with complete WD were found (Table 1). These proteins belonged to three main WRKY groups and their subgroups, which were distributed throughout the genome (Fig 1). Seven members presented variations in the WD (in the heptapeptide or in the C_2_H_2_ zinc finger motifs), suggesting a higher divergence, possibly due to recent mutations, of these genes in comparison to the rest of the TcWRKY family. Groups IIc and III (5 and 1 gene, respectively) contained 70% of the amino acid variations observed, suggesting that these two groups were more subjected to selective pressure and variability through time. Analysis in other species such as cotton or tomato, also showed that these two groups were the most divergent in the evolutionary history of the WRKY family [53, 54]. The phylogenetic analysis revealed that subgroups IIa and IIb are sister groups and share a common ancestor, as well as subgroups IId and IIe (Fig 4). Various studies have demonstrated that the expansion of WRKY TF family members is mainly due to gene duplication events, as shown in rice [55], *Arabidopsis* [56], cotton [53], *Populus* [57] and barley [58]. Here, we observed that 40% of the *TcWRKY* sequences presented one or more duplication events (Fig 3) and that these events were associated mainly with the conservation of the TcWRKY motif patterns (Figs 4 and 5). Generally, the duplicated genes were also present together in the same clades of the motif phylogeny as observed for Tc04_g029800/Tc01_g031960, Tc04_g009710/Tc02_g032670, Tc03_g028700/Tc03_g017550/Tc01_g034680, Tc03_g009820/Tc02_g033950, Tc03_g028030/Tc03_g019750/Tc01_g035330, Tc01_g014750/Tc06_g013130, Tc07_g002910/Tc06_g019530, Tc09_g034740/Tc05_g001480, Tc04_g004210/Tc03_g015140 and Tc08_g000030/Tc01_g005580 (Figs 3, 4 and 5). However, in the case of the duplication of Tc08_g013540/Tc01_g010370, the two sequences were located in different phylogenetic clades, suggesting an evolution of the gene and motif structure (Figs 3 and 4). Phylogenetic analysis in macromolecules, by forming non-random clusters, also suggests that these molecules may share the same biological functions, may be present in the same cell compartment or be expressed/produced at the same moment during a biological process. Besides the heptapeptide WRKYGQK and the C_2_H_2_/C_2_HC zinc-finger motifs–known to be involved in DNA-binding–some TcWRKY proteins (Tc02_g001230, Tc02_g001200, Tc02_g001170 and Tc00_g017270) showed conserved motifs, such as NB-ARC and/or LRR (Fig 5C), known to be involved in pathogen recognition, plant resistance and activation of plant immunity [59, 60].

Expression and functional analysis of WRKY TFs could help in discriminating the role and function of these proteins at the tissue and organism levels. Here, we evaluated by RT-qPCR the expression of seven *TcWRKY* genes in resistant and susceptible cacao plants inoculated or not with *M*. *perniciosa*. The choice of the genes was based on previous indications in the genome databank (CocoaGenDB), phylogenetic analysis and putative function of the orthologues in *Arabidopsis*, showing that: i) Tc01_p014750 and Tc06_p013130 (indicated as *TcWRKY28*) were co-orthologous to *AtWRKY8* and *AtWRKY28*; ii) Tc09_p001530 and Tc06_p004420 (both indicated as *TcWRKY40*) were both orthologous to *AtWRKY40*; iii) Tc04_p016130 and Tc10_p016570 were co-orthologous to *AtWRKY54* and *AtWRKY70*; and iv) Tc01_p018460 was orthologous to *AtWRKY1* (Fig 4). Interestingly, the Tc01_p014750 and Tc06_p013130 genes came from an event of duplication (Fig 3) but showed different expression patterns (Fig 6B), suggesting that sequence evolution may result in different roles and/or functions in relation to pathogen response. These two sequences were co-orthologous to *AtWRKY*8 and *AtWRKY28*, which are induced by ABA, wounding, oxalic acid (OA) and/or hydrogen peroxide (H_2_O_2_) [61, 62]. In cacao, it has been reported that the amount of calcium oxalate crystal (COC) and H_2_O_2_ levels in the TSH1188 (resistant) *vs*. Catongo (susceptible) varieties present distinct temporal and genotype dependent patterns [40, 41]: susceptible variety accumulated more COC than the resistant one, and the COC dissolution–resulting in OA and H_2_O_2_ formation–occurred in the early infection stages in the resistant variety and in the final stage of the disease in the susceptible one. Interestingly, the *Tc01_p014750* gene, orthologous to *AtWRKY28*, showed higher expression in the early infection stages in TSH1188 and 15 dai in the Catongo variety (Fig 6B), stages during which the H_2_O_2_ is considered the highest [40, 41]. The *Tc06_p013130* gene (previously annotated as *TcWRKY28*) showed an expression pattern different from Tc01_p014750, with high abundance 24 hai in the susceptible variety and a constant and low abundance in the resistant one (Fig 6). Such divergent behavior was previously observed for the rice *WRKY28* gene. Delteil et al. [63] reported that the knock-out of *OsWRKY28* by T-DNA insertion leads to a two-fold increase in resistance to a compatible rice blast fungus, and this phenotype is accompanied with increased expression of several defense-related genes. Likewise, other authors showed that the overexpression of *OsWRKY28* resulted in enhanced susceptibility to the rice blast fungus *Magnaporthe oryzae* and decreased accumulation of PR-5 [64]. According to the authors, these phenotypes observed in overexpression or genetic defects in *OsWRKY28* are consistent with their presumed role as negative regulators of basal defense responses to compatible rice blast fungus strains [64]. The same role was also suggested for *WRKY8* in *Arabidopsis*. This gene could be a negative or positive regulator of the basal resistance of the plant when infected by *Pseudomonas syringae* or *Botrytis cinerea*, respectively [61]. In cacao, it can be suggested that Tc01_p014750 acted as a positive regulator of plant resistance to *M*. *perniciosa* through activation by OA and/or reactive oxygen species (ROS); the involvement of OA and ROS in cacao resistance to *M*. *perniciosa* has been previously observed in studies using the same or similar plant genotypes and culture conditions [40, 41, 65]. On the other hand, Tc06_p013130 may have acted as negative regulator of the basal resistance of cacao.

The Tc09_p001530 and Tc06_p004420 genes (both indicated as *TcWRKY40*) showed high transcript abundance in the susceptible cacao variety (final stages; Fig 6B). In *Arabidopsis*, studies have shown that the WRKY18, WRKY40 and WRKY60 TFs are induced by pathogens and interact physically and functionally together forming homo and heterocomplexes [66]. The constitutive overexpression of these genes in *Arabidopsis* increased its susceptibility to *B*. *cinerea* [66]. Moreover, the superexpression of WRKY40 in transgenic *Populus trichocarpa* plants conferred high susceptibility to the hemibiotrophic fungus *Dothiorella gregaria* Sacc., indicating that PtrWRKY40 plays a negative role in resistance to this hemibiotrophic fungus in poplar [67]. In cacao, the Tc09_p001530 and Tc06_p004420 genes (*TcWRKY40*) may have a similar function to that observed in poplar: the gene expression in the Catongo variety may be associated with the plant susceptibility to *M*. *perniciosa*. In the phylogenetic analysis, the sequences Tc04_p016130 and Tc10_p016570 were grouped both with AtWRKY54 and AtWKY70 (Fig 4). Tc04_p016130 and Tc10_p016570 showed similar expression patterns, mainly with a very high expression in the last time point in TSH1188 (about 10 and 140 times more at 45 dai, respectively). Several works have reported the cooperation of the *AtWRKY54* and *AtWKY70* genes in response to biotic and abiotic stresses [68, 69]. These TFs are positive regulators of plant defense, and cooperate as negative regulators of salicylic acid (SA) biosynthesis and senescence [69], but are not responsive to signals such as ROS [68]. The WRKY70 TF was identified as an integrator in cross-talk between SA and jasmonic acid (JA), two hormones with a well-defined function in plant defense response regulation [70]. Generally, SA is associated with defense response against biotrophic pathogens, whereas JA has a function in defense responses against herbivore and necrotrophic pathogens [70]. In cacao, previous works have shown an increase of jasmonate biosynthesis genes in the last time stages in TSH1188-*M*. *perniciosa* interaction (from 30 to 60 dai), as well as an increase of ROS detoxification genes [39]. The gene Tc01_p018460 showed high phylogenetic proximity with AtWRKY1 but also with other genes involved in pathogen responses induced by SA, such as AtWRKY3 and ATWRKY58 (Fig 4); this proximity may be related to similar gene function. In the susceptible genotype, the *Tc01_p018460* expression was higher mainly 7 dai (Fig 6B).

## Conclusion

Here, we identified 61 WRKY proteins from *T*. *cacao*, distributed on all the chromosomes, in some cases coming from different duplication events. To our knowledge, this is the first report of the entire WRKY TF family in cacao and of expression analysis in relation to *M*. *perniciosa* infection. The TcWRKY family showed a phylogenetic composition similar to that of *Arabidopsis* and some couple of sequences showed similar expression patterns and possibly functions (e.g., Tc01_p014750/Tc06_p013130/AtWRKY28; Tc09_p001530/Tc06_p004420/AtWRKY40; Tc04_p016130/*AtWRKY54*; Tc10_p016570/*AtWRKY70*; Tc01_p018460/*AtWRKY1*). Mainly, the *Tc04_p016130* and *Tc10_p016570* sequences presented a special interest due to their high and differential expression level between resistant and susceptible plants infected by *M*. *perniciosa*. In general, our results can help to select appropriate candidate genes for further characterization and/or confirmation studies in relation to pathogen resistance in cacao or in other *Theobroma* species, as well as for support of future breeding efforts.

## Supporting information

S1 Fig General scheme of the *in silico* pipeline used for identification and confirmation of the TcWRKY sequences. (DOCX)

S2 Fig Disease symptoms observed in the TSH1188 (resistant) and Catongo (susceptible) infected and non-infected plants. White arrow: swelling of the stem; black arrow: ramification (green broom).
(DOCX)

S1 Table List of the 153 *T*. *cacao* and *A*. *thaliana* WRKY domains used for phylogeny. (DOCX)

S2 Table Primers used in this study. (DOCX)

S3 Table Characteristics of WRKY amplicons. (DOCX)

## Abbreviations

ABA: abscisic acid
COC: calcium oxalate crystals
hai: hours after inoculation
dai: days after inoculation
JA: jasmonic acid
LRR: leucine-rich repeat
OA: oxalic acid
ROS: reactive oxygen species
SA: salicylic acid
TF: transcription factor
WD: WRKY domain
