# Supplementary material for: Genome-wide identification and characterization of cacao WRKY transcription factors and analysis of their expression in response to witches' broom disease
Source: PLoS One. 2017 Oct 30;12(10):e0187346. doi: 10.1371/journal.pone.0187346 (PMC5662177; doi:10.1371/journal.pone.0187346)

**S1 Figure.** General scheme of the *in silico* pipeline used for identification and confirmation of the TcWRKY sequences.


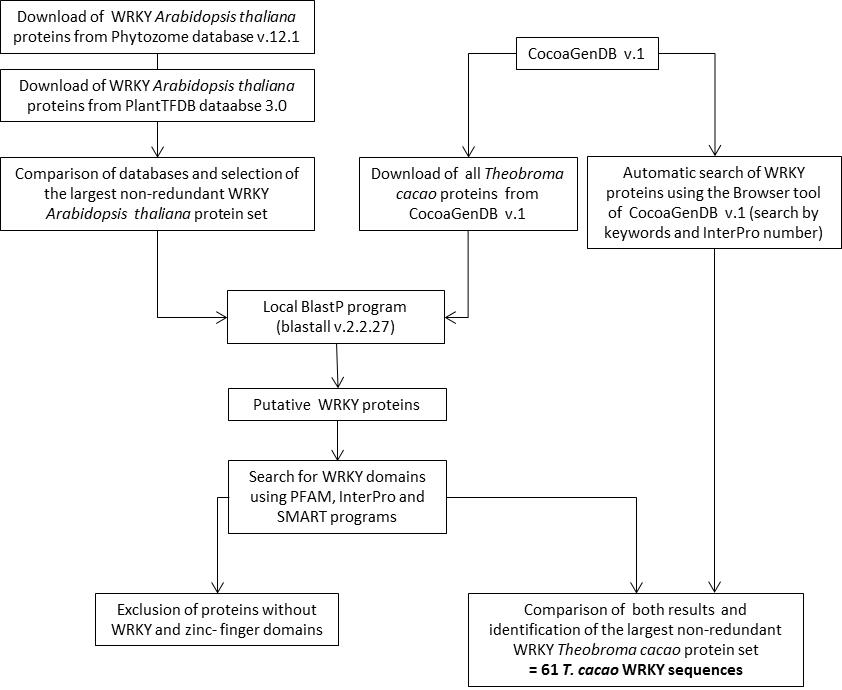

Supplement: S1 Fig — (DOCX) [file pone.0187346.s001.docx]
