# Supplementary material for: Genome-wide identification and characterization of cacao WRKY transcription factors and analysis of their expression in response to witches' broom disease
Source: PLoS One. 2017 Oct 30;12(10):e0187346. doi: 10.1371/journal.pone.0187346 (PMC5662177; doi:10.1371/journal.pone.0187346)

**S2 Figure.** Disease symptoms observed in the TSH1188 (resistant) and Catongo (susceptible) inoculated and non-inoculated plants. White arrow: swelling of the stem; black arrow: ramification (green broom).


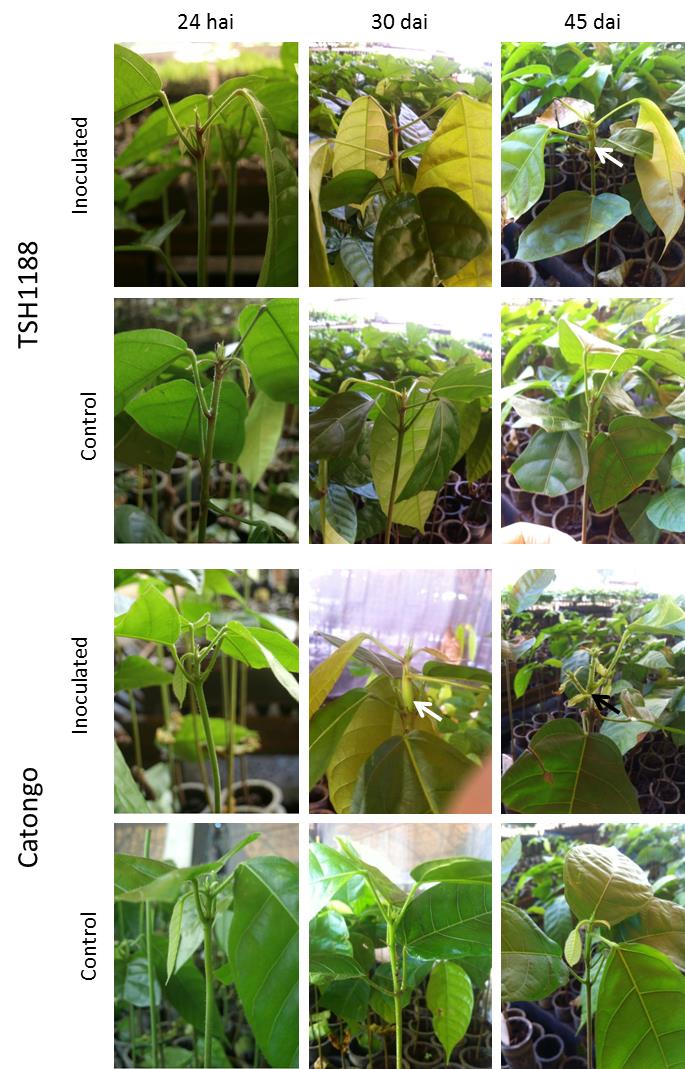

Supplement: S2 Fig — White arrow: swelling of the stem; black arrow: ramification (green broom). (DOCX) [file pone.0187346.s002.docx]
