# Supplementary material for: Genome-wide identification and characterization of cacao WRKY transcription factors and analysis of their expression in response to witches' broom disease
Source: PLoS One. 2017 Oct 30;12(10):e0187346. doi: 10.1371/journal.pone.0187346 (PMC5662177; doi:10.1371/journal.pone.0187346)
Supplement: S1 Table — (DOCX) [file pone.0187346.s003.docx]

**S1 Table.** List of the 153 *T. cacao* and *A. thaliana* WRKY domains used for phylogeny.

| >Tc00_g017240 | VKSRKSDDAYSWRCIGIKGLIGNRRRSFYKCARPGFPATKSVDRSLDGQITEIIHKARHNHPES |
| --- | --- |
| >Tc09_g002780N | IVKTPVSDGYNWRKYGQKQVKSPKGSRSYYKCTFSDCRAKKIECSDHTGHVIEIVNKGMHSHEPPRKHNLT |
| >AtWRKY32N | VPRTPARDGYNWRKYGQKQVKSPKGSRSYYRCTYTECCAKKIECSNDSGNVVEIVNKGLHTHEPP |
| >Tc01_g018460N | VREKASEDGYNWRKYGQKLVKGNEFVRSYYKCTHPNCLVKKQLERSHDGKMVDTVYFGQHDHPKPLNLPVA |
| >AtWRKY1N | IREKVMEDGYNWRKYGQKLVKGNEFVRSYYRCTHPNCKAKKQLERSAGGQVVDTVYFGEHDHPKP |
| >AtWRKY44N | TGDRSSVDGYNWRKYGQKQVKGSECPRSYYKCTHPKCPVKKKVERSVEGQVSEIVYQGEHNHSKP |
| >Tc05_g005710N | NIDRPSYDGYNWRKYGQKQVKGSEYPRSYYKCTHPNCPVKKKVERSFDGQIAEIVYKGEHNHSKPQPPKRN |
| >AtWRKY19N | NVDKQVNDGYNWQKYGQKKVKGSKFPLSYYKCTYLGCPSKRKVERSLDGQVAEIVYKDRHNHEPP |
| >AtWRKY3N | NADKPADDGYNWRKYGQKQVKGSDFPRSYYKCTHPACPVKKKVERSLDGQVTEIIYKGQHNHELP |
| >Tc02_g032670N | IVDKPADDGYNWRKYGQKQVKGSEFPRSYYKCTHPGCPVKKKVERSLDGQVTEIIYKGQHNHQPPQSNKR |
| >AtWRKY4N | NVDKPADDGYNWRKYGQKQVKGSEFPRSYYKCTNPGCPVKKKVERSLDGQVTEIIYKGQHNHEPP |
| >Tc04_g009710N | AVDKPAEDGYNWRKYGQKPIKGCEYPRSYYKCTHLNCPVKKKVERSADGQITEIIYKGAHNHEKPQPNKQG |
| >AtWRKY58N | NVDKPADDGYNWRKYGQKPIKGCEYPRSYYKCTHVNCPVKKKVERSSDGQITQIIYKGQHDHERP |
| >AtWRKY25N | MVSRNSNDGYGWRKYGQKQVKKSENPRSYFKCTYPDCVSKKIVETASDGQITEIIYKGGHNHPKP |
| >Tc07_g002020N | GPSVPSDDGYNWRKYGQKQVKGSEFPRSYYKCTHPNCEVKKLFERSHDGQITEIIYKGTHDHPKPQPSRRY |
| >AtWRKY20N | TPSILADDGYNWRKYGQKHVKGSEFPRSYYKCTHPNCEVKKLFERSHDGQITDIIYKGTHDHPKP |
| >AtWRKY26N | SSNKTSDDGYNWRKYGQKQVKGSENPRSYFKCTYPNCLTKKKVETSLVKGQMIEIVYKGSHNHPKP |
| >AtWRKY33N | REQRKGEDGYNWRKYGQKQVKGSENPRSYYKCTFPNCPTKKKVERSLEGQITEIVYKGSHNHPKP |
| >Tc09_g034740N | RENRRSDDGYNWRKYGQKQVKGSENPRSYYKCTFPNCPTKKKVERSLDGQITEIVYKGSHNHPKPQSTRRS |
| >Tc05_g001480N | RENRKVQDGYNWRKYGQKQVKGSENPRSYYKCTYPNCPTKKKVERSLDGQITEIVYKGSHNHPKPQSTRRS |
| >AtWRKY34N | ACCAPADDGYNWRKYGQKLVKGSEYPRSYYKCTHPNCEAKKKVERSREGHIIEIIYTGDHIHSKP |
| >Tc05_g020810N | GMARTSEDGYNWRKYGQKQVKGSEYPRSYYKCTHPNCQVKKKVERSLDGQITEIIYKGAHNHPKPLPCRRP |
| >Tc07_g000190N | VGSAPSDDGYNWRKYGQKQVKGSEYPRSYYKCTHPNCQVKKKVERSHEGHITEIIYKGAHNHPKPPPNRRS |
| >AtWRKY2N | AGGAPAEDGYNWRKYGQKLVKGSEYPRSYYKCTNPNCQVKKKVERSREGHITEIIYKGAHNHLKP |
| >AtWRKY36 | CEDPSINDGCQWRKYGQKTAKTNPLPRAYYRCSMSSNCPVRKQVQRCGEEETSAFMTTYEGNHDHPLP |
| >AtWRKY59 | DEKVALDDGYKWRKYGKKPITGSPFPRHYHKCSSPDCNVKKKIERDTNNPDYILTTYEGRHNHPSP |
| >Tc02_g001230 | GLGEPLPDGYNWRKHGQTDIVGARYPRTYYRCAHLLTVGCLATKQVQRVDENPMIFSVTYSGKHTCNLASD |
| >Tc02_g001200 | VLGELPSDGCNWRKYGQKDILNARFPREYYRCAHRHTQGCFATKEVQREDEDPMFITATYKGMHTCTLAPD |
| >Tc10_g016560 | NTDLPPEDNFTWRKYGQKEILGSRYPRAYYRCTHQKLYNCPAKKQVQRLDNDFYTFEVTYIGQHTCHMSST |
| >AtWRKY55 | NTDLPPDDNHTWRKYGQKEILGSRFPRAYYRCTHQKLYNCPAKKQVQRLNDDPFTFRVTYRGSHTCYNS |
| >AtWRKY46 | QENGSIDDGHCWRKYGQKEIHGSKNPRAYYRCTHRFTQDCLAVKQVQKSDTDPSLFEVKYLGNHTCNNI |
| >AtWRKY30 | GVDRTLDDGFSWRKYGQKDILGAKFPRGYYRCTYRKSQGCEATKQVQRSDENQMLLEISYRGIHSCSQA |
| >AtWRKY41 | GLEGPHDDIFSWRKYGQKDILGAKFPRSYYRCTFRNTQYCWATKQVQRSDGDPTIFEVTYRGTHTCSQG |
| >AtWRKY53 | GLEGPQDDVFSWRKYGQKDILGAKFPRSYYRCTHRSTQNCWATKQVQRSDGDATVFEVTYRGTHTCSQA |
| >Tc03_g028700 | LLEGPHDDGYSWRKYGQKDILGAKYPRSYYRCTYRNTQNCWATKQVQRSDEDPAIFEITYRGTHTCALGNQ |
| >Tc03_g017550 | ALEGPLDDGFSWRKYGQKDILGAKYPRGYYRCTHRNVQGCLATKQVQRSDDDPTIFEITYRGRHTCTVASH |
| >Tc01_g034680 | SLEGPLDDGYCWRKYGQKDILGSNFPRGYYRCTHRHSQGCLATKQVQRSDEDPTIFEVKYRGRHTCNQVSH |
| >AtWRKY52 | IPAIDEGDLWTWRKYGQKDILGSRFPRGYYRCAYKFTHGCKATKQVQRSETDSNMLAITYLSEHNHPRP |
| >AtWRKY38 | SPDPIYYDGYLWRKYGQKSIKKSNHQRSYYRCSYNKDHNCEARKHEQKIKDNPPVYRTTYFGHHTCKTE |
| >AtWRKY62 | SSTPIYHDGFLWRKYGQKQIKESEYQRSYYKCAYTKDQNCEAKKQVQKIQHNPPLYSTTYFGQHICQLH |
| >AtWRKY66 | SPTPAHIDGFIWRKYGQKTIKTSPHQRWYYRCAYAKDQNCDATKRVQKIQDNPPVYRNTYVGQHACEAP |
| >AtWRKY67 | SRTMCPNDGFTWRKYGQKTIKASAHKRCYYRCTYAKDQNCNATKRVQKIKDNPPVYRTTYLGKHVCKAF |
| >AtWRKY64 | SPTPRPDDGFTWRKYGQKTIKTSPYQRCYYRCTYAKDQNCNARKRVQMIQDNPPVYRTTYLGKHVCKAV |
| >AtWRKY63 | SPNPRLDDGFTWRKYGQKTIKTSLYQRCYYRCAYAKDQNCYATKRVQMIQDSPPVYRTTYLGQHTCKAF |
| >Tc04_g016130 | DSPTLIDDGHAWRKYGQKVILNAKHPRNYYRCTHKHDQGCQATKQVQQIEDDPPKYGTTYYGHHTCKNLLK |
| >Tc10_g016570 | VISAAMEDGHAWRKYGQKEILNAKHPRSYFRCTRKYDQGCRATKQVQRMEDDSQMFQTVYIGSHTCRDSSK |
| >AtWRKY54 | VEAKSSEDRYAWRKYGQKEILNTTFPRSYFRCTHKPTQGCKATKQVQKQDQDSEMFQITYIGYHTCTAN |
| >AtWRKY70 | IESTILEDAFSWRKYGQKEILNAKFPRSYFRCTHKYTQGCKATKQVQKVELEPKMFSITYIGNHTCNTN |
| >AtWRKY21 | KVADIPPDDYSWRKYGQKPIKGSPYPRGYYKCSSMRGCPARKHVERCLEDPAMLIVTYEAEHNHPKL |
| >AtWRKY39 | KIADIPPDEYSWRKYGQKPIKGSPHPRGYYKCSSVRGCPARKHVERCIDETSMLIVTYEGEHNHSRI |
| >AtWRKY74 | KIADIPPDEYSWRKYGQKPIKGSPHPRGYYKCSSVRGCPARKHVERCVEETSMLIVTYEGEHNHSRI |
| >Tc05_g027100 | KVADIPPDEYSWRKYGQKPIKGSPHPRGYYKCSSVRGCPARKHVERCLEDPSMLIVTYEGEHNHSRLLSTQ |
| >Tc01_g027130 | KLADIPPDDYSWRKYGQKPIKGSPHPRGYYKCSSMRGCPARKHVERCLEEPSMLIVTYEGEHNHPRLPSQS |
| >Tc09_g000780 | KIADIPPDEFSWRKYGQKPIKGSPYPRGYYKCSTIRGCPARKHVERAPDDPAMLIVTYEGEHRHSQPAMQD |
| >AtWRKY17 | KIADIPPDEYSWRKYGQKPIKGSPHPRGYYKCSTFRGCPARKHVERALDDSTMLIVTYEGEHRHHQS |
| >AtWRKY11 | KIADIPPDEYSWRKYGQKPIKGSPHPRGYYKCSTFRGCPARKHVERALDDPAMLIVTYEGEHRHNQS |
| >Tc03_g025390 | KLSDIPPDDYSWRKYGQKPIKGSPYPRSYYKCSSMRGCPARKHVERCLEDPTMLVVTYEGDHKHSRITFQP |
| >AtWRKY7 | KMADIPSDEFSWRKYGQKPIKGSPHPRGYYKCSSVRGCPARKHVERALDDAMMLIVTYEGDHNHALV |
| >AtWRKY15 | KMSDVPPDDYSWRKYGQKPIKGSPHPRGYYKCSSVRGCPARKHVERAADDSSMLIVTYEGDHNHSLS |
| >Tc08_g000030 | KMADIPPDDFSWRKYGQKPIKGSPHPRGYYKCSSVRGCPARKHVERAVDDPRMLIVTYEGDHNHSHNITDA |
| >Tc01_g005580 | KMADIPPDDYSWRKYGQKPIKGSPHPRGYYKCSSVRGCPARKHVERALDDPSMLIVTYEGEHNHPLSLAET |
| >Tc01_g031780 | KNEGPPSDLWSWRKYGQKPIKGSPYPRGYYRCSTSKGCSAKKQVERCRTDASMLIITYTSSHNHPGPDLHT |
| >AtWRKY16 | DRGSRSSDLWVWRKYGQKPIKSSPYPRSYYRCASSKGCFARKQVERSRTDPNVSVITYISEHNHPFP |
| >AtWRKY14 | SGEVVPSDLWAWRKYGQKPIKGSPFPRGYYRCSSSKGCSARKQVERSRTDPNMLVITYTSEHNHPWP |
| >Tc06_g000970 | SGEVVPSDLWAWRKYGQKPIKGSPYPRGYYRCSSSKGCSARKQVERSRTDPNMLVITYTSEHNHPWPTQRN |
| >AtWRKY35 | SGEVVPSDLWAWRKYGQKPIKGSPYPRGYYRCSSSKGCSARKQVERSRTDPNMLVITYTSEHNHPWP |
| >AtWRKY65 | GDTTPPSDSWAWRKYGQKPIKGSPYPRGYYRCSSTKGCPARKQVERSRDDPTMILITYTSEHNHPWP |
| >AtWRKY69 | GEVYPPSDSWAWRKYGQKPIKGSPYPRGYYRCSSSKGCPARKQVERSRVDPSKLMITYACDHNHPFP |
| >Tc06_g013990 | GESAPPSDSWAWRKYGQKPIKGSPYPRGYYRCSSSKGCPARKQVERSRMDPSMLVITYSCEHNHPWPASRN |
| >AtWRKY29 | KEENLLSDAWAWRKYGQKPIKGSPYPRSYYRCSSSKGCLARKQVERNPQNPEKFTITYTNEHNHELP |
| >AtWRKY22 | AAEALNSDVWAWRKYGQKPIKGSPYPRGYYRCSTSKGCLARKQVERNRSDPKMFIVTYTAEHNHPAP |
| >Tc01_g035330 | PAEGLSADVWAWRKYGQKPIKGSPYPRGYYRCSSSKGCLARKQVERNRSDPAMFIVTYTAEHNHPAPTHRN |
| >Tc03_g019750 | AVDNLSSDPWAWRKYGQKPIKGSPYPRNYYRCSSSKGCAARKQVERSNFDPNIFIVTYTGDHTHPRPTHRN |
| >AtWRKY27 | TQENLSSDLWAWRKYGQKPIKGSPYPRNYYRCSSSKGCLARKQVERSNLDPNIFIVTYTGEHTHPRP |
| >Tc03_g028030 | TADGLSSDIWAWRKYGQKPIKGSPYPRSYYRCSSSKGCLARKQVERSCSDPRIFIITYTAEHSHGHPTRRS |
| >Tc09_g001520 | DKSLIVKDGFQWRKYGQKVTKDNPSPRAYFRCSMAPGCPVKKKVQRCVEDKSFLLATYEGQHNHDVHSSPM |
| >AtWRKY60 | DTSLTVKDGYQWRKYGQKITRDNPSPRAYFRCSFSPSCLVKKKVQRSAEDPSFLVATYEGTHNHTGP |
| >AtWRKY18 | DTSLTVKDGFQWRKYGQKVTRDNPSPRAYFRCSFAPSCPVKKKVQRSAEDPSLLVATYEGTHNHLGP |
| >Tc09_g001530 | DNSLIVRDGYQWRKYGQKVTRDNPSPRAYFKCSFAPSCPVKKKVQRSAEDPSILVATYEGEHNHAHSSPAE |
| >AtWRKY40 | DTTLVVKDGYQWRKYGQKVTRDNPSPRAYFKCACAPSCSVKKKVQRSVEDQSVLVATYEGEHNHPMP |
| >Tc06_g004420 | DTSLVVKDGYQWRKYGQKVTRDNPSPRAYFKCSFAPSCPVKKKVQRSVEDQLVLVATYEGEHNHLPPSQME |
| >AtWRKY47 | SDATTVNDGCQWRKYGQKMAKGNPCPRAYYRCTMAVGCPVRKQVQRCAEDTTILTTTYEGNHNHPLP |
| >Tc07_g002910 | SEASMISDGCQWRKYGQKMAKGNPCPRAYYRCTMATGCPVRKQVQRCADDRTILNTTYEGNHNHPLPPAAM |
| >AtWRKY31 | SEAAMISDGCQWRKYGQKMAKGNPCPRAYYRCTMAGGCPVRKQVQRCAEDRSILITTYEGNHNHPLP |
| >AtWRKY6 | SEAPMISDGCQWRKYGQKMAKGNPCPRAYYRCTMATGCPVRKQVQRCAEDRSILITTYEGNHNHPLP |
| >Tc01_g032940 | SEAPLISDGCQWRKYGQKMAKGNPCPRAYYRCTMAVGCPVRKQVQRCAEDKSILITTYEGNHNHPLPPAAT |
| >AtWRKY42 | SEAPMLSDGCQWRKYGQKMAKGNPCPRAYYRCTMAVGCPVRKQVQRCAEDRTILITTYEGNHNHPLP |
| >Tc06_g019530 | SEAPMITDGCQWRKYGQKMAKGNPCPRAYYRCTMAVGCPVRKQVQRCAEDRTILITTYEGNHNHPLPPAAM |
| >Tc01_g017430 | SEAPMITDGCQWRKYGQKMAKGNPCPRAYYRCTMAAGCPVRKQVQRCAEDRTILITTYEGNHNHPLPPAAM |
| >Tc02_g033950 | CDTPTLNDGCQWRKYGQKIAKGNPCPRAYYRCTVSPTCPVRKQVSVQRCAEDMSILITTYEGNHNHPLPLS |
| >Tc04_g007790 | CDAPTMNDGCQWRKYGQKISKGNPCPRAYYRCTVAPGCPVRKQVDSCLVAKVQRCAEDMSILITTYEGNHNHP |
| >Tc02_g003250 | CQTATMNDGCQWRKYGQKIAKGNPCPRAYYRCTVAPGCPVRKQVQRCLEDMSILITTYEGTHNHPLPVGAT |
| >AtWRKY9 | CETATMNDGCQWRKYGQKTAKGNPCPRAYYRCTVAPGCPVRKQVQRCLEDMSILITTYEGTHNHPLP |
| >AtWRKY61 | CETPTMNDGCQWRKYGQKIAKGNPCPRAYYRCTIAASCPVRKQVQRCSEDMSILISTYEGTHNHPLP |
| >AtWRKY72 | CDTPTMNDGCQWRKYGQKIAKGNPCPRAYYRCTVAPGCPVRKQVQRCADDMSILITTYEGTHSHSLP |
| >Tc03_g009820 | CDTPTMNDGCQWRKYGQKIAKGNPCPRAYYRCTVAPSCPVRKQVQRCAEDMSILITTYEGTHNHPLPMSAT |
| >Tc05_g004380 | CGNGMADDGYKWRKYGQKSIKNSPNPRSYYKCTNPRCSAKKQVERSRDDPDTLIITYEGLHLHFPYPYFSL |
| >AtWRKY49 | NSNGMCDDGYKWRKYGQKSIKNSPNPRSYYKCTNPICNAKKQVERSIDESNTYIITYEGFHFHYTY |
| >Tc00_g076580 | TNIELLDDGYKWRKYGKKQVKGNPNSRNYYKCSTVACPVKKRVERDPLDTRYLITTYEGMHNHESPFVKAL |
| >Tc00_g017270 | TNIELVDDGYKWRKYGKKQVKGNPNSRNYYKCSTELCPVKKRVERDHLDTRYLISTYDGIHNHERPFVKAL |
| >Tc08_g013540 | SDLEIMDDGYKWRKYGKKRVKNNPNPRNYYRCSTAGCKVKKRVERDKEDPRFVITTYEGKHHHESPSADTD |
| >Tc09_g005290 | SEVEILDDGYRWRKYGKKMVKNSPNPRNYYRCSVEGCTVKKRVERDREDPSYVVTTYEGIHNHQSAS |
| >AtWRKY51 | SKIDVMDDGFKWRKYGKKSVKNNINKRNYYKCSSEGCSVKKRVERDGDDAAYVITTYEGVHNHESL |
| >AtWRKY50 | SEVEVLDDGFKWRKYGKKMVKNSPHPRNYYKCSVDGCPVKKRVERDRDDPSFVITTYEGSHNHSSM |
| >Tc01_g010220 | SELEVMDDGYKWRKYGKKSVKNSPNPRNYYKCSSGGCNVKKRIERDRDDTSYVITTYDGIHNHDSPYMVYY |
| >AtWRKY56 | SDDDVLDDGYRWRKYGQKSVKNNAHPRSYYRCTYHTCNVKKQVQRLAKDPNVVVTTYEGVHNHPCE |
| >AtWRKY24 | SDDDVLDDGYRWRKYGQKSVKHNAHPRSYYRCTYHTCNVKKQVQRLAKDPNVVVTTYEGVHNHPCE |
| >AtWRKY43 | SDADILDDGYRWRKYGQKSVKNSLYPRSYYRCTQHMCNVKKQVQRLSKETSIVETTYEGIHNHPCE |
| >Tc01_g035290 | SADDILDDGYRWRKYGQKAVKNSNYPRSYYRCTHHTCNVKKQVQRLSKDTSIVVTTYEGIHNHPCEKLMET |
| >AtWRKY45C | SQVDILDDGYRWRKYGQKAVKNNPFPRSYYKCTEEGCRVKKQVQRQWGDEGVVVTTYQGVHTHAVD |
| >Tc03_g015140 | SQVDILDDGYRWRKYGQKTVKNSKFPRSYYRCTHKECNVKKQVQRSSKDDEIVVTTYEGIHTHPVEKFTEN |
| >AtWRKY75 | SQVDILDDGYRWRKYGQKAVKNNKFPRSYYRCTYGGCNVKKQVQRLTVDQEVVVTTYEGVHSHPIE |
| >Tc00_g047270 | SQVDILDDGYRWRKYGQKAVKNNKFPRSYYRCTHQGCNVKKQVQRLTRDEGIVVTTYEGMHSHPIQKSNDN |
| >Tc04_g004210 | SQVDILDDGYRWRKYGQKAVKNNKFPRSYYRCTHQGCNVKKQVQRLTKDESVVVTTYEGMHTHPIEKSTDN |
| >AtWRKY12 | SDVDVLDDGYKWRKYGQKVVKNSLHPRSYYRCTHNNCRVKKRVERLSEDCRMVITTYEGRHNHIPS |
| >Tc01_g039500 | SDVDVLDDGYKWRKYGQKVVKNSLHPRSYYRCTHNNCRVKKRVERLSEDCRMVITTYEGRHNHSPCDDSNS |
| >Tc01_g010370 | SDVDVLDDGYKWRKYGQKVVKNTQHPRSYYRCTQDNCRVKKRVERLAEDPRMVITTYEGRHVHSPSHDLED |
| >AtWRKY13 | SEVDVLDDGYRWRKYGQKVVKNTQHPRSYYRCTQDKCRVKKRVERLADDPRMVITTYEGRHLHSPS |
| >Tc04_g029800 | SEVDHLDDGYRWRKYGQKAVKNSPYPRSYYRCTSAGCGVKKRVERSSDDPTVVVTTYEGQHTHPCPITPRG |
| >AtWRKY48 | SDIDNLDDGYRWRKYGQKAVKNSPYPRSYYRCTTVGCGVKKRVERSSDDPSIVMTTYEGQHTHPFP |
| >Tc01_g014750 | SEIDHLEDGYRWRKYGQKAVKNSPYPRSYYRCTSQKCTVKKRVERSFQDPSVVITTYEGQHNHHIPATLRG |
| >AtWRKY71 | SEIDHLEDGYRWRKYGQKAVKNSPYPRSYYRCTTQKCNVKKRVERSFQDPSIVITTYEGKHNHPIP |
| >AtWRKY8 | TEVDHLEDGYRWRKYGQKAVKNSPYPRSYYRCTTQKCNVKKRVERSYQDPTVVITTYESQHNHPIP |
| >Tc06_g013130 | SEVDHLEDGYRWRKYGQKAVKNSPYPRSYYRCTTQKCTVKKRVERSFQDPSVVITTYEGQHNHPLPTTLRG |
| >AtWRKY28 | SEVDHLEDGYRWRKYGQKAVKNSPYPRSYYRCTTQKCNVKKRVERSFQDPTVVITTYEGQHNHPIP |
| >Tc02_g032350 | SEVDHLEDGYRWRKYGQKAVKNSPYPRSVAISKSEKTDMSYYRCTNSKCTVKKRVERSSEDPTTVITTYEGQHCHHS |
| >AtWRKY57 | SDVDNLEDGYRWRKYGQKAVKNSPFPRSYYRCTNSRCTVKKRVERSSDDPSIVITTYEGQHCHQTI |
| >AtWRKY68 | SEVLHLDDGYKWRKYGQKPVKDSPFPRNYYRCTTTWCDVKKRVERSFSDPSSVITTYEGQHTHPRP |
| >Tc01_g031960 | SEVDHLEDGYRWRKYGQKAVKNSPFPRSYYRCTTTSCNVKKRVERSFSDPSIVVTTYEGQHTHPSPVIPRP |
| >AtWRKY23 | SEVDHLEDGYRWRKYGQKAVKNSPFPRSYYRCTTASCNVKKRVERSFRDPSTVVTTYEGQHTHISP |
| >Tc09_g002780C | GDVGISGDGYRWRKYGQKMVKGNPNPRNYYRCTSAGCPVRKHIETAVDNTNAVIITYKGVHDHDMPVPKKR |
| >AtWRKY32C | GDVGICGDGYRWRKYGQKMVKGNPHPRNYYRCTSAGCPVRKHIETAVENTKAVIITYKGVHNHDMP |
| >AtWRKY44C | VESDSLEDGFRWRKYGQKVVGGNAYPRSYYRCTSANCRARKHVERASDDPRAFITTYEGKHNHHLL |
| >Tc05_g005710C | TDSEIMGDGFRWRKYGQKVVKGNPYPRSYYRCTSLKCNVRKHVERASDDPRAFITTYEGKHNHEMPLRNTN |
| >AtWRKY1C | TLFDIVNDGYRWRKYGQKSVKGSPYPRSYYRCSSPGCPVKKHVERSSHDTKLLITTYEGKHDHDMP |
| >Tc01_g018460C | SEVDIVNDGYRWRKYGQKLVKGNPNPRSYYRCSNPGCPVKKHVERDSHDVKLVITTYEGRHDHDIPPTRTV |
| >AtWRKY10C | SDEDNPNDGYRWRKYGQKVVKGNPNPRSYFKCTNIECRVKKHVERGADNIKLVVTTYDGIHNHPSP |
| >AtWRKY25C | SDIDVLIDGFRWRKYGQKVVKGNTNPRSYYKCTFQGCGVKKQVERSAADERAVLTTYEGRHNHDIP |
| >AtWRKY34C | SDIDILDDGYRWRKYGQKVVKGNPNPRSYYKCTANGCTVTKHVERASDDFKSVLTTYIGKHTHVVP |
| >AtWRKY58C | SEVDLLDDGYRWRKYGQKVVKGNPHPRSYYKCTTPNCTVRKHVERASTDAKAVITTYEGKHNHDVP |
| >AtWRKY4C | SEVDLLDDGYRWRKYGQKVVKGNPYPRSYYKCTTPGCGVRKHVERAATDPKAVVTTYEGKHNHDLP |
| >AtWRKY3C | SEVDLLDDGYRWRKYGQKVVKGNPYPRSYYKCTTPDCGVRKHVERAATDPKAVVTTYEGKHNHDVP |
| >Tc04_g009710C | SEVDLLDDGYRWRKYGQKVVKGNPHPRSYYKCTSAGCNVRKHVERASTDPKAVITTYEGKHNHDVPAARNS |
| >Tc02_g032670C | SEVDLLDDGYRWRKYGQKVVKGNPYPRSYYKCTTPGCNVRKHVERASTDPKAVITTYEGKHNHDVPAAKTS |
| >AtWRKY26C | SDIDILDDGYRWRKYGQKVVKGNPNPRSYYKCTFTGCFVRKHVERAFQDPKSVITTYEGKHKHQIP |
| >Tc05_g020810C | SDVDILDDGYRWRKYGQKVVKGNPNPRSYYKCTTPGCPVRKHVERASHNLKCVLTTYDGKHNHEVPAARSS |
| >Tc09_g034740C | SDIDILDDGYRWRKYGQKVVKGNPNPRSYYKCTHPGCPVRKHVERASHDRRAVITTYEGKHNHDVPAARGS |
| >Tc05_g001480C | SDIDILDDGYRWRKYGQKVVKGNPNPRSYYKCTTIGCPVRKHVERASHDLRAVITTYEGKHNHDVPAARGS |
| >AtWRKY33C | SDIDILDDGYRWRKYGQKVVKGNPNPRSYYKCTTIGCPVRKHVERASHDMRAVITTYEGKHNHDVP |
| >AtWRKY2C | SDVDILDDGYRWRKYGQKVVKGNPNPRSYYKCTAPGCTVRKHVERASHDLKSVITTYEGKHNHDVP |
| >Tc07_g000190C | SEVDILDDGYRWRKYGQKVVKGNPNPRSYYKCTSAGCTVRKHVERASHDLKSVITTYEGKHNHDVPAARSS |
| >AtWRKY20C | SEVDILDDGYRWRKYGQKVVRGNPNPRSYYKCTAHGCPVRKHVERASHDPKAVITTYEGKHDHDVP |
| >Tc07_g002020C | SEVDILDDGYRWRKYGQKVVRGNPNPRSYYKCTNAGCPVRKHVERASHDPKAVITTYEGKHNHDVPTARTS |
