# Supplementary material for: Genome-wide identification and characterization of cacao WRKY transcription factors and analysis of their expression in response to witches' broom disease
Source: PLoS One. 2017 Oct 30;12(10):e0187346. doi: 10.1371/journal.pone.0187346 (PMC5662177; doi:10.1371/journal.pone.0187346)
Supplement: S2 Table — (DOCX) [file pone.0187346.s004.docx]

**S2 Table.** Primers used in this study.

| Gene | Primer 5’-3’ | Size (bp) | %GC | Tm (°C) | Reference |
| --- | --- | --- | --- | --- | --- |
| Tc04_t016130 | F: TTCTCAGCTTTCCCACCAGT | 20 | 50 | 59.84 | This study |
|  | R: TTGAGCTGATGACTCGAACG | 20 | 50 | 60.14 |  |
| Tc10_t016570 | F: TGCTAAGCACCCAAGGAGTT | 20 | 50 | 59.88 |  |
|  | R: TGGATGAGTCTCTGCAGGTG | 20 | 55 | 59.98 |  |
| Tc09_t001530 | F: GGGTTAGCGCTGAGAACAAG | 20 | 55 | 60.02 |  |
|  | R: AGTTCTTGCTCACCAATTCCA | 21 | 42.86 | 59.73 |  |
| Tc06_t004420 | F: ATGGGTGGATACTTCGTTGG | 20 | 50 | 59.67 |  |
|  | R: TGCTAGTCGACTCCTTCTTCG | 21 | 52.38 | 59.77 |  |
| Tc06_t013130 | F: TACTACCAGGCTGCATTCCA | 20 | 50 | 59.30 |  |
|  | R: AACAATCCGTGAAGCTCATGT | 21 | 42.86 | 59.61 |  |
| Tc01_t014750 | F: CAAGGATTCAACCGATCGAC | 20 | 50 | 60.46 |  |
|  | R: TTTTGGGTTGGAGCTGTTAGA | 21 | 42.86 | 59.73 |  |
| Tc08_t013540 | F: CACCACCTTCCTAGCAGCAG | 20 | 60 | 60.98 |  |
|  | R: CATGACGGCGTGACTGTAAA | 20 | 50 | 60.72 |  |
| Tc01_t018460 | F: TATGGTTTCTTCGGGGGAGT | 20 | 50 | 60.68 |  |
|  | R: CCTCCTTGATTGGATTGTGG | 20 | 50 | 60.31 |  |
| GAPDH | F: GATGCTCCTATGTTTGTTGTGG | 22 | 45.45 | 54 | Pinheiro et al. (2012)  Menezes et al. (2014) |
|  | R: TCTTCCTCCTCTCCAGTCCTT | 21 | 52.38 | 57 |  |
| MDH | F: AAAATGGAGTTGGTGGATGC | 20 | 45 | 54 |  |
|  | R: AACCATGACTGCGATGTTGA | 20 | 45 | 55 |  |
