# Supplementary material for: Genome-wide identification and characterization of cacao WRKY transcription factors and analysis of their expression in response to witches' broom disease
Source: PLoS One. 2017 Oct 30;12(10):e0187346. doi: 10.1371/journal.pone.0187346 (PMC5662177; doi:10.1371/journal.pone.0187346)
Supplement: S3 Table — (DOCX) [file pone.0187346.s005.docx]

**S3 Table.** Characteristics of WRKY amplicons

| Gene | Tm°C | CG% | Size (bp) | A | T | G | C | GC/AT |
| --- | --- | --- | --- | --- | --- | --- | --- | --- |
| Tc04_t016130 | 95 | 46.7 | 137 | 42 | 31 | 43 | 21 | 0.87 |
| Tc10_t016570 | 95 | 45.9 | 146 | 50 | 29 | 36 | 31 | 0.84 |
| Tc09_t001530 | 91.6 | 44.1 | 102 | 34 | 23 | 18 | 27 | 0.789 |
| Tc06_t004420 | 92.1 | 51.3 | 80 | 19 | 20 | 18 | 23 | 1.05 |
| Tc06_t013130 | 92.5 | 43.1 | 109 | 28 | 34 | 23 | 24 | 0.758 |
| Tc01_t014750 | 92.4 | 45.7 | 92 | 28 | 22 | 30 | 12 | 0.84 |
| Tc08_t013540 | 93.9 | 52.4 | 82 | 24 | 15 | 31 | 12 | 1.102 |
| Tc01_t018460 | 85.5 | 42 | 69 | 21 | 19 | 9 | 20 | 0.725 |
